# Traumatic brain injury related deaths in residents and non-residents of 30 European countries: a cross-sectional study

**DOI:** 10.1038/s41598-023-34560-7

**Published:** 2023-05-10

**Authors:** Patrik Sivco, Dominika Plancikova, Juliana Melichova, Martin Rusnak, Iva Hereitova, Vaclav Beranek, Roman Cibulka, Marek Majdan

**Affiliations:** 1 Institute for Global Health and Epidemiology, Department of Public Health, Faculty of Health Sciences and Social Work, Trnava University, Hornopotocna 23, 91843 Trnava, Slovakia; 2 Department of Rehabilitation Studies, Faculty of Health Care Studies, University of West Bohemia, 30100 Pilsen, Czech Republic; 3 Department of Paramedic Science, Medical Diagnostics Studies and Public Health, University of West Bohemia, 30100 Pilsen, Czech Republic

**Keywords:** Brain injuries, Trauma

## Abstract

The incidence and mortality of traumatic brain injuries (TBI) among non-residents to countries where they occur remains unknown, warranting epidemiological research. Epidemiological data are key to inform prevention and public health policies related to TBI, as well as to help promote safe travelling practice. The aim of this study was to analyse the epidemiological patterns of TBI-related deaths among residents and non-residents in 30 European countries in 2015 using standardised European level data on causes of death. A large-scale cross-sectional study analysing TBI-related deaths in 30 European countries in 2015 among residents and non-residents to the country of occurrence of the death was conducted. Data from death certificates collected on European level by Eurostat were used to calculate the numbers of TBI-related deaths and estimate crude and age-standardised mortality rates. Rates were stratified by country, sex, age-group and by resident status. External causes of the injury were determined using the provided ICD-10 codes. 40,087 TBI-related deaths were identified; overall about 3% occurred among non-residents with highest proportions in Turkey (11%), Luxembourg (9%) and Cyprus (5%). Taking into account tourism intensity in the countries, Bulgaria, Greece and Austria showed highest rates of TBI-related deaths in non-residents: 0.7,0.5 and 0.5 per million overnight stays, respectively. The pooled age-standardised TBI-related mortality in non-residents was 0.2 (95% CI 0.1–0.3), among residents 10.4 (95% CI 9.4–11.5) per 100,000. In non-residents, TBI-related deaths were shifted to younger populations (86% in < 35 years); in non-residents 78% were 15–64 years old. Falls were predominant among residents (47%), and traffic accidents among non-residents (36%). Male:female ratio was higher among non-residents (3.9), compared to residents (2.1). Extrapolating our findings, we estimate that annually 1022 TBI-related deaths would occur to non-residents in the EU-27 + UK and 1488 in Europe as a continent. We conclude, that the primary populations at risk of TBI-related deaths in European countries differ in several characteristics between residents and non-residents to the country of the occurrence of death, which warrants for different approaches in prevention and safety promotion. Our findings suggest that TBI occurring in European countries among non-residents present a problem worthy of attention from public health and travel medicine professionals and should be further studied.

## Introduction

Europe has been the most visited tourist region in the world, welcoming more than half of the total international arrivals in 2015 (51% of all international arrivals, over 600 million in total), with almost all European destinations reporting an increase in foreign visits and visitor nights in comparison to previous years^1^. Furthermore, according to Eurostat, 31% of the EU-28 inhabitants stayed in a foreign country for personal purposes for at least one night in 2015^2^. Thus, EU is a region with amongst the highest international travel intensity in the world^1^.

Injuries are a substantial public health problem: according to the Global Burden of Diseases study, about 973 million inuries occurred globally in 2013, resulting in about 4.8 million deaths^3^. In Europe, a relatively large variation has been observed, especially in terms of injury-related deaths: in 2019 the injury-related mortality was 80/100,000 in Eastern Europe which is twice as high as in Central Europe (38/100,000) and three times higher compared to Western Europe (27/100,000)^3^.

Besides being an important cause of death in general, injuries have also been shown to be a major cause of death among international travellers^4–6^. A study estimated that around 30% of all deaths to travellers in Europe are caused by injuries^7^. While cardiovascular diseases (such as myocardial infarction or stroke) are the most common cause of death in travellers^8^, the public health importance of injuries as a cause of death lies in their preventability by policy measures imposed in countries or by adjusting individual behaviour and risk taking. Furthermore, multiple reports have shown that when compared to domestic populations, travellers appear to have a higher risk of injury mortality with relative rates of injury death ranging from 1.04 to 16.7 per 100,000^9^.

Of all injuries, traumatic brain injuries (TBI) are the major cause of disability and deaths, burdening individuals, families, health systems and societies^10^. A recent study estimated that TBI caused about 57,000 deaths and were the reason for nearly 1.5 million hospital admissions in countries of the EU^11^. It has also been reported that TBI-related deaths have been associated with a substantial number of years of lost life (YLL)—on average, each TBI-related death in the EU was associated with about 24 YLLs, totalling to about 1.3 million YLLs attributable to TBI in 2013^12^. Overall, about 37% of all injury-related deaths in Europe are caused by a TBI^11^ and TBI contribute about 44% to all injury related YLLs^12^. TBI is not isolated to specific countries and cause major life losses among the children, productive population, and the elderly in high-income as well as low-income countries^10,13^.

The main causes of injuries among travellers are relatively well documented in published studies, highlighting traffic accidents as the primary cause, followed by drownings and homicides^6–9,14^. On the other hand, studies that analyse the nature of injuries leading to traveller fatalities (E.g., which body regions were injured) are largely lacking in the literature, including those focusing on TBI. To the best of our knowledge, the only epidemiological study that analysed the incidence and mortality of TBI in residents and visitors is a study from Austria which reported a higher incidence of TBI hospital admissions among foreigners (727/100,000) compared to residents (292/100,000). According to their findings, most cases were caused by falls and were likely related to winter sports, as 75% of TBI in foreign nationals occurred during winter or spring^15^.

Thus, the burden of TBI among non-residents to countries where they occur remains unknown, warranting epidemiological research into their causes, occurrence, and outcomes. Such data are of key importance to inform preventative action, public health policies, and health care needs across the EU related to TBI, as well as to help promote safe travelling practice. With this study we are filling this gap and presenting a comprehensive European-wide analysis of the epidemiological patterns of TBI in non-residents compared to residents. Furthermore, we used standardised data sources that allow for valid between-country comparisons, largely overcoming the substantial variability of studies analysing data from single countries, which use different sources of data, case definitions or approaches to analysis^9,14^.

The aim of this study was to analyse the epidemiological patterns of TBI-related deaths among residents and non-residents in 30 European countries in 2015 using standardised European level data on causes of death.

## Methodology

### Study design and population

A cross-sectional epidemiological study has been conducted using data on causes of death from 30 European countries in 2015 (Austria, Belgium, Bulgaria, Cyprus, Czechia, Germany, Denmark, Estonia, Finland, Greece, Croatia, Hungary, Switzerland, Ireland, Island, Italy, Lithuania, Luxembourg, Latvia, Malta, Netherlands, Norway, Portugal, Romania, Serbia, Sweden, Slovenia, Slovakia, Turkey, and United Kingdom were included). Countries were included based on the availability of data, i.e., their participation in the European surveillance system for causes of deaths. The year 2015 was the latest year with reported and available data at the time of study analysis.

### Data sources

All data analysed in the study were provided as micro-level data by the Statistical Office of the European Union (Eurostat)^16,17^ upon a special request (data in such detail is not being included in regular reports on causes of death from Eurostat). The data on causes of deaths are based on death certificates and are being collected by Eurostat from European countries in accordance with a European regulation^18^. Eurostat performs a validity check on all data submitted by countries. This process ensures a high level of validity and cross-country comparability of all data on causes of death. The provided dataset contained records of all injury-related deaths in 2015 provided to Eurostat by countries, including information on the external cause of injury and the nature of injury [both coded using the 10th revision of the International Classification of Diseases (ICD-10)], information on the sex and age of the deceased, and the country where the death occurred. In addition, information on whether the deceased was a resident or a non-resident to the country where the death occurred has been provided for each death. Due to changes in the practice of data provision at Eurostat, no data in the needed detail could be provided for the study for any year beyond 2015, despite multiple requests.

Data on the number of overnight stays in countries by incoming foreign visitors were obtained from the tourism statistics database of Eurostat^2^. Mid-year population counts for 2015 used to recalculate the numbers of deaths to the population of the respective countries were obtained from Eurostat population database^19^. Data on the number of overnight stays for countries were obtained from the tourism statistics database of Eurostat^2^.

### Variable definitions

For the purposes of this study, TBI-related deaths were defined using ICD-10 codes—codes ranging from S00 to S09 (injuries to the head) and T90 (sequelae of injuries to the head) were considered TBI. The external causes of injury were grouped into five categories: traffic accidents (ICD-10 external causes of injury codes V01-V99), falls (W00-W19), suicide (X60-X84), violence (X85-Y09) and other causes (W20-X59 and Y10-Y98). Deaths were counted for each country and stratified by residence status at the time of death to ‘residents’ (E.g., those having a permanent residence to the country where the death occurred, at time of death) and ‘non-residents’ (E.g., those not having a permanent residence to the country where the death occurred, at the time of death), cause of injury, sex, and age using the following age-groups: 0–4, 5–14, 15–34, 35–64, 65–84, and 85 years old or older.

Crude mortality rates per 100,000 and age-standardised mortality rates per 100,000 person-years with 95% confidence intervals were calculated for each stratum and overall, for each country. The 2013 revision of the European standard population^20^ was used to calculate the age-standardised mortality rates (using the direct method of standardisation).

To show the frequency of TBI-related deaths in non-residents in relation to the intensity of tourism in the analysed countries, number of TBI-related deaths per 1 million overnight stays by incoming foreigners were calculated.

### Statistical methods

Pooled crude and age-standardised mortality rates (crude and age-standardised) were calculated applying the random-effects model (25), using the DerSimonian and Laird method (26). The heterogeneity of the country estimates was assessed using I^2^ with 95% confidence intervals. As the study used population wide data, no p-values were calculated. Extrapolations of the observed pooled rates were done by recalculation these rates to the population counts of the EU (both EU-27 and EU-27 plus the UK) and Europe as a continent.

### Ethical approval and consent to participate

The study used publicly available administratively collected data and ethical approval was not sought. All methods in the presented study were performed in accordance with the relevant guidelines and regulations.

## Results

### Overall TBI-related deaths in the analysed countries

In the 30 analysed countries in 2015, 40,087 cases of death were identified as TBI-related according to the study’s case-definition; of these, 39,004 (97%) occurred among residents of the included countries and 1083 (3%) among the non-residents. The highest proportions of TBI-related deaths among non-residents were observed in Turkey (11%), Luxembourg (9%), and Cyprus (7%) (See Supplementary Table S1 for details). TBI-related deaths in the analysed countries were more prevalent in men (27,389 deaths; 68%) compared to women (12,676 deaths; 32%). The ratio of males to females was 2.1 in residents, 3.9 in non-residents and 2.2 in all cases combined.

The pooled age-standardised TBI-related mortality rate in residents and non-residents combined for the 30 analysed countries was estimated at 10.6 per 100,000 person-years (95% CI 9.7–11.8), with highest rates observed in Finland and lowest in Malta. The highest observed mortality rate among men was in Lithuania (32.6 per 100,000 population; 95% CI 29.3–36.1) and among women in Finland (10.4 per 100,000; 95% CI 9.3–11.6)—see Supplementary Figs. S1–S3 for details.

### TBI-related deaths in residents versus non-residents

**Figure 1:**
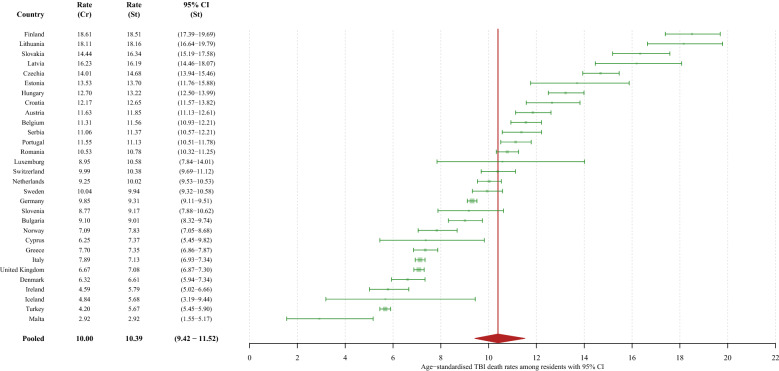
Crude TBI-related mortality rates and age-standardised TBI-related mortality rates per 100,000 person-years among residents of 30 European countries in 2015, with a pooled age-standardised mortality rate. Heterogeneity assessment: I^2^ = 99.4% (95% CI 99.4% to 99.5%). St: standardised; CI: confidence interval; Cr: crude.

Among residents, the pooled age-standardised TBI-related mortality rate was estimated at 10.4 per 100,000 (95% CI 9.4–11.5). The rank of countries remained the same for men, women, and both sexes combined (See Fig. 1 and Supplementary Figures S4 and S5 for details).

**Figure 2:**
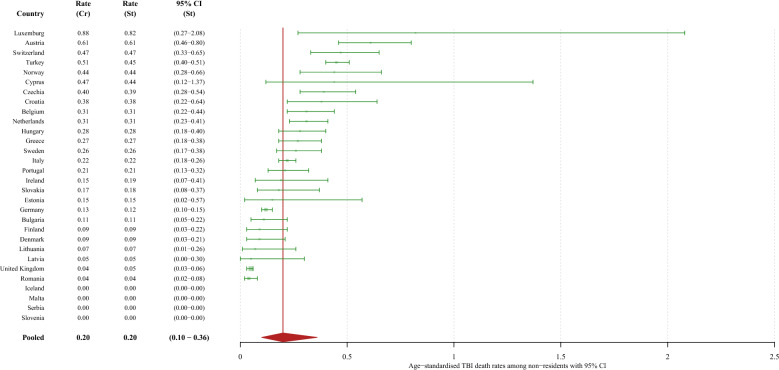
Crude TBI-related mortality rates and age-standardised TBI-related mortality rates per 100,000 person-years in 30 European countries among non-residents in 2015, with a pooled age-standardised mortality rate. Heterogeneity assessment: I^2^ = 95.4% (95% CI 94.3% to 96.3%). St: standardised; CI: confidence interval; Cr: crude.

The pooled age-standardised TBI-related mortality rate among non-residents was estimated to be 0.2 per 100,000 population (95% CI 0.1–0.4) for both sexes combined (0.3 per 100,000; 95% CI 0.1–0.7 in men and 0.1 per 100,000; 95% CI 0.01–0.2 in women). The countries with the highest rates overall were Luxembourg, Austria, Switzerland, Turkey, and Norway, while Malta, Serbia, Iceland and Slovenia recorded no TBI-related deaths among non-residents in 2015. See Fig. 2 and Supplementary Figures S6 and S7 for details.

**Figure 3:**
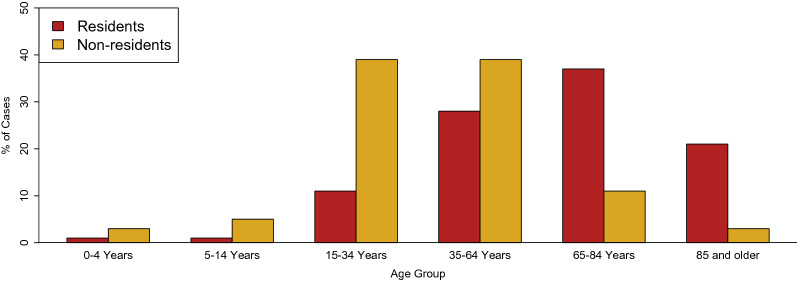
Distribution of TBI-related deaths in residents and non-residents in 30 European countries in 2015 by age-groups.

Regarding the ages of the deceased, the observed patterns between residents and non-residents differed. In residents, most deaths occurred in the age group of 65–84 years old (14,548, 37%), followed by the age group of 35–64 years old (11,007, 28%) whereas among the non-residents most TBI-related deaths occurred in the younger populations: 425 (39%) among the 35–64 years old and 419 (38%) among the 15–34 years old. Thus, a shift of TBI-related deaths to younger populations is observed among non-residents, compared to residents. Please see Fig. 3 for a graphical display of these distributions, and Supplementary Tables S2—S4 for detailed analyses stratified by country and sex.

**Figure 4:**
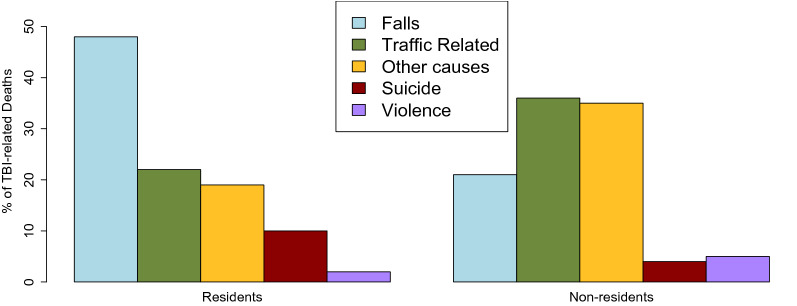
Distribution of TBI-related deaths in residents and non-residents in 30 European countries in 2015 by cause of death.

The main causes of injury leading to TBI-related deaths differed between residents and non-residents. Overall, the most frequent external cause of injury were falls (18,705 deaths; 47%) followed by traffic-related causes (8,838 deaths; 22%) with violence being the least prevalent cause (983; 2% of deaths). Differences in the distribution of causes were observed between residents and non-residents: among residents, the most prevalent external cause were falls (47%), followed by traffic-related causes (22%), while among non-residents, traffic related causes were predominant (36%), with falls being the cause in 21% of deaths and over a third of deaths had a cause recorded as ‘other causes’. Please see Fig. 4 for a detailed comparison Supplementary Tables S5–S7 for detailed calculations (percentages, crude rates, and age-standardised rates) stratified by country and sex.

In both, residents and non-residents combined, the highest age-standardised TBI-related mortality rate due to falls was observed in Finland (11.5 per 100,000 (95% CI 10.6–12.4), in case of traffic accidents the highest rate was observed in Romania (3.8 per 100,000; 95% CI 3.6–4.1), for suicide in Finland (2.7 per 100,000; 95% CI 2.3–3.2), and for violence in Latvia (1.5 per 100,000; 95% CI 1.0–2.1). When looking at non-resident populations separately, for falls the highest mortality rate was observed in Austria (0.3 per 100,000; 95% CI 0.2–0.5), for traffic accidents the highest mortality rate was observed in Luxembourg (0.5 per 100,000; 95% CI 0.1–1.6), and for suicide in the Netherlands (0.1 per 100,000; 95% CI 0.02–0.1). Detailed distributions stratified by sex are shown in Supplementary Tables S6 and S7.

**Figure 5:**
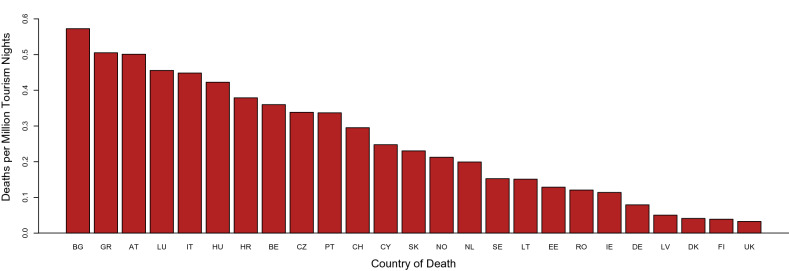
TBI-related deaths among non-residents per 1 million overnight stays by incoming foreign visitors to European countries in 2015. AT, Austria; BE, Belgium; BG, Bulgaria; CY, Cyprus; CZ, Czechia; DE, Germany; DK, Denmark; EE, Estonia; FI, Finland; GR, Greece; HR, Croatia; HU, Hungary; CH, Switzerland; IE, Ireland; IS, Island; IT, Italy; LT, Lithuania; LU, Luxemburg; LV, Latvia; MT, Malta; NL, Netherlands; NO, Norway; PT, Portugal; RO, Romania; RS, Serbia; SE, Sweden; SI, Slovenia; SK, Slovakia; TR, Turkey; UK, United Kingdom.

To better show the occurrence of TBI-related deaths in context of the intensity of international travelling to the analysed countries, Fig. 5 shows a country comparison in the number of TBI-related deaths in non-residents recalculated to a million overnight stays by incoming foreign visitors. Using these calculations, on average 0.16 TBI-related deaths occurred per one million overnight stays in the analysed countries. The highest rates were observed in Bulgaria (0.57 deaths per 1 million overnight stays), Greece and Austria (both 0.5 deaths per million), while for Denmark, Finland, or UK the values were substantially lower (0.04, 0.03 and 0.03, respectively). Relatively large between-country variations were observed.

### Extrapolation to EU and Europe as a continent

To show the magnitude of the problem from the perspective of the EU and Europe as a continent, we extrapolated the observed age-standardised TBI-related death rates in non-residents to the populations of these regions. Based on this extrapolation, we estimated that in the EU (27 countries, excluding the UK) about 891 (95% CI 446–1603) deaths due to TBI would have occurred annually between 2015 and 2019, while in the EU including the population of the UK 1022 (95% CI 511–1840), and in Europe as a continent 1488 (95% CI 744–2678) such deaths would have occurred among non-residents to countries where these deaths occurred (See Supplementary Table S8 for details). Table 1 summarizes all major findings to give a clear overview of all calculated parameters and an overview of all observed differences in TBI-related deaths between the two compared groups.

**Table 1:** Summary of demographic characteristics, external causes, and crude and age-standardised TBI related mortality rates among residents and non-residents of 30 European countries in 2015.

Analysed Parameters	Residents N = 39,004 (97%)		Non-residents N = 1083 (3%)	
Sex (N, % Males)	26,543 (68%)		846 (80%)	
Age (N, %)				
0–4 years	370 (1%)		28 (%)	
5–14 years	390 (1%)		54 (5%)	
15–34 years	4377 (11%)		419 (39%)	
35–64 years	11,007 (28%)		425 (39%)	
65–84 years	14,548 (37%)		123 (11%)	
85 + years	8312 (21%)		34 (3%)	
External Cause (N, %)				
Falls	18,479 (47%)		226 (21%)	
Suicide	3783 (10%)		44 (4%)	
Violence	933 (2%)		50 (5%)	
Traffic	8450 (22%)		388 (36%)	
Other	7359 (19%)		375 (35%)	

Crude TBI-related mortality rates (Per 100,000)	Pooled Estimate	Range	Pooled Estimate	Range
Both sexes	10.00	(2.92–18.61)	0.20	(0.00–0.88)
Females	5.78	(1.35–12.29)	0.07	(0.00–0.35)
Males	14.51	(4.48–29.37)	0.31	(0.00–1.40)

Age-standardised TBI-related mortality rates (Per 100,000)	Pooled Estimate (95% CI)	Range	Pooled Estimate (95% CI)	Range
Both sexes	10.39 (9.42–11.52)	(2.92–18.51)	0.20 (0.10–0.36)	(0.00–0.82)
Females	5.48 (4.55–6.71)	(1.26–10.40)	0.06 (0.01–0.18)	(0.00–0.34)
Males	16.57 (14.75–18.85)	(4.61–32.40)	0.31 (0.14–0.71)	(0.00–1.27)

## Discussion

### Main findings

We conducted a large-scale cross-sectional study analysing TBI-related deaths in 30 European countries in 2015 among residents and non-residents to the country of occurrence of the death. Overall, 40,087 TBI-related deaths were identified, of which about 3% occurred among non-residents. Countries with the highest proportion of non-resident deaths were Turkey (11% of all TBI-related deaths), Luxembourg (9%) and Cyprus (5%). Recalculated to reflect the intensity of tourism, Bulgaria, Greece and Austria had the highest rates of TBI-related deaths: 0.7, 0.5 and 0.5 per million overnight stays, respectively. The pooled age-standardised TBI-related mortality rate in non-residents in the analysed countries was 0.2 (95% CI 0.1–0.3), while among residents the rate was 10.4 (95% CI 9.4–11.5). The following key differences between residents and non-residents were observed: (1) in non-residents, TBI-related deaths were shifted to younger populations (in residents, 86% of deaths occurred in 35 years or older, while 78% of deaths in non-residents occurred in 15–64 years old); (2) while falls were the predominant cause among residents (47% of deaths), traffic accidents were predominant in non-residents (36%); (3) males were substantially more predominant among non-residents (male to female ratio of 3.9), compared to residents (male to female ratio of 2.1). By extrapolating the observed pooled age-standardised TBI-related mortality rate, we estimate that annually about 891 TBI-related deaths would occur to non-residents in the EU-27, while about 1022 such deaths would occur in the EU-27 + the UK and 1488 in the population of Europe as a continent.

### Comparison to other studies and interpretation

To the best of our knowledge, this is the first study to analyse TBI-related deaths in residents versus non-residents on this scale and thus presents a unique set of findings. In general, the published literature on this topic is extremely limited, therefore, the possibility to directly compare our findings to other studies is restricted.

Previous studies have investigated TBI-related mortality in Europe on a broader than the national scale, but none of them was aimed at the resident or non-resident population specifically. For the general population, European cross-sectional studies (using the same case definition and statistical approach) reported a pooled age-standardised TBI mortality rate of 11.7 per 100,000 in 2012^11^ and 11.3 per 100,000 in 2013^12^, with higher mortality rates in men compared to women. Our study yielded a very similar age-standardised rate of 10.6 per 100,000 population—the slight difference may be explained by the true change in the TBI-related mortality in Europe, but also by including a slightly different set of countries for the analyses. Thus, in general, our findings are similar to previous reports, which confirms the robustness and validity of our analyses.

In our study, the age-standardised mortality rate among residents to countries where the deaths occurred was estimated at 10.4 per 100,000. This can be directly compared to the findings from TBI surveillance in the US which for 1997–2007 estimated the TBI-related death rate among US residents at 18.4 per 100,000 (range: 17.8–19.3)^21^—the higher mortality rate may be caused by general differences in the population or life-style between Europe and the US, with some key factors such as large numbers of firearm-related TBI deaths^22^ being possible drivers.

The only study to which our findings are directly comparable is the study of Mauritz et al.^15^ that analysed hospital admissions and deaths due to TBI among residents and non-residents in Austria. Among visitors, the study estimated the TBI-related mortality in males at 0.9 per 100,000 and in females at 0.7 per 100,000. In our study the rates at 0.3 per 100,000 in males and 0.06 per 100,000 in females which is relatively lower, possibly due to the larger scale of the study and the more substantial variation in the analysed population thereof. On the other hand, the mortality rate for non-residents in Austria in our study has been estimated at 0.61 (in 2015), which is reasonably similar to the estimations for 2009–2011^15^ and support the validity of the findings presented in this paper.

In general, when reflecting the intensity of international travelling in the countries, non-residents in Bulgaria, Greece and Austria were at highest risk of TBI-related deaths of all 30 countries (0.5 or more TBI-related deaths per 1 million overnight stays). While this study does not present evidence, we can hypothesize that the relatively high rate in Austria may be associated with the fact that Austria is a major destination for winter sports. This has been pointed out in the study of Mauritz et al. from Austria which concludes that most of the registered cases of TBI in non-residents may have been associated with winter sports^15^. In our study, in European countries that are traditional destinations for winter sports such as Austria or Switzerland the proportion of non-resident TBI-related deaths was relatively high (around 5%, see Supplementary Table S1 for details), which may support this hypothesis. However, further studies should investigate this in more detail to provide more evidence. Regarding the high risk observed in Bulgaria or Greece, we could hypothesize that these are driven by traffic accidents—based on our findings in both countries the observed mortality rates due to traffic accidents were higher compared to mortality rates associated with falls or other causes (a pattern that is reversed in most of the countries analysed in this paper, consult Supplementary Table S7). However, further studies should analyse this in more detail.

Our study identified key differences between the demographic characteristics of non-residents and residents dying as consequence of TBI. First, the age structure of non-residents is shifted towards younger age-groups, compared to residents (see Fig. 3 and Table 1), which corresponds with the finding of the study from Austria where lower mean age was observed among non-residents compared to residents (28 years vs. 28 years)^15^. While these findings may reflect true differences in the age structure of TBI victims between the two compared groups, they may also be biased by the fact that the population of non-residents in European countries tends to be younger than the population of residents^23^. Secondly, non-residents in our study were prevailingly men. The generally higher risk of TBI-related death in males compared to females is a well-documented fact^10,13,21^; however, our findings suggest that in case of non-residents the male to female ratio is nearly two-fold compared to the male to female ratio in residents (2.1 in residents vs. 3.9 in non-residents). This finding is in line with a higher proportion of males among tourist fatalities in Europe, which was estimated at up to 80%^24,25^. In general, the higher risk of injury incidence and mortality in males can be attributed in part to higher engagement in risk taking behaviour compared to females, as documented in the literature^26,27^. In summary, the population of non-residents dying as a consequence of TBI in Europe is younger and has a higher proportion of males, compared to residents dying due to TBI. These differences should be observed by public health and travel medicine professionals to better target prevention. Further epidemiological research is needed to elucidate in more detail the causes and drivers of TBI incidence and mortality among non-residents. Targeted action, such as more detailed surveillance implemented by European or national authorities is also warranted and may help to prevent avoidable morbidity and mortality caused by TBI by informing targeted and tailored prevention.

### Limitations and generalizability

The data analysed in this study comes from 30 European countries, where some practices in the process of certification of deaths and assigning causes of death may differ, eventually introducing selection bias. However, the data was provided to us by Eurostat which oversees its aggregation on the European level, and the fact that the certification of deaths in the EU is governed by an EU regulation (No 328/2011)^18^ ensures the highest level of validity and between-country comparability of these data that is possible under the circumstances. The data used for the analyses in this study comes from a single year of 2015 and thus no trends could be analysed and comparisons between multiple years were not possible. We are aware of this limitation and made all efforts to obtain data for further years in the needed detail, but this was not possible. Furthermore, 2015 was the most recent year for which we were able to obtain the data needed for the study and may not reflect the true epidemiological situation at the time of publication of this study. However, previous studies looking at TBI-related mortality in general populations^7,10,13^ (E.g., not split to residents vs. non-residents) using the same data sources and multiple consecutive years showed little variation in injury characteristics (such as sex, causes, age structure) between the years. This suggests that the findings regarding the structure of residents versus non-residents among the countries would not vary substantially under normal circumstances and could be generalizable to other years as well.

TBI for the purposes of this study was defined using the full range of codes included in the chapter of “Injuries to the head” of the ICD-10 (E.g., S00-S09) and the code for sequelae of injuries to the head (E.g., T90), including a variety of injuries of diverse severities. We have considered all deaths where the primary cause was coded using one of these codes to be TBI-related deaths and included them in the study. We are aware that some of the codes denote “head injuries” and do not directly imply a TBI. However, we have chosen this strategy for the following reasons: (1) previous research has shown that more narrow case definitions may lead to underestimation of the actual incidence or mortality of TBI in population level studies using administrative data sources^28,29^; (2) previous studies also suggest that for analyses focusing on epidemiology and prevention, a broader definition of TBI is advisable (as opposed to studies looking at clinical characteristics and outcome where the case definition should be more strict)^28^; and (3) the same case definition has been used by previous European-wide studies of TBI epidemiology and burden^11,12^, which creates grounds for better comparability of findings across all studies, and allows for bringing the main findings of this study into context with the overall epidemiological patterns of TBI incidence and mortality in Europe. Furthermore, we note that when broken down into specific diagnostic groups, most of the deaths (72%) were coded as intracranial injuries (ICD-10 code S06), about 10% of cases were coded S02 (fracture of skull and facial bones), about 11% were coded S09 (Other and unspecified injuries of head), with the remaining 7% of cases distributed among the other diagnostic groups within the case definition (see Supplementary Table S9 for details). Such distribution suggests the case definition used in the study was robust and valid. On the other hand, we note that the findings of our study should be interpreted with caution and with these characteristics in mind.

## Conclusions

Primary populations at risk of TBI-related deaths in European countries differ in several characteristics between residents and non-residents to the country of the occurrence of death and warrant for different approaches in prevention and safety promotion. Our findings suggest that TBI occurring in European countries among non-residents present a problem worthy of attention from public health and travel medicine professionals and should be further studied.

## Supplementary Information

Supplementary Information.

## Data Availability

The datasets used and/or analyzed during the current study available from the corresponding author on reasonable request.
