# Supplementary material for: Traumatic brain injury related deaths in residents and non-residents of 30 European countries: a cross-sectional study
Source: Sci Rep. 2023 May 10;13:7610. doi: 10.1038/s41598-023-34560-7 (PMC10172191; doi:10.1038/s41598-023-34560-7)
Supplement: Supplementary file 1 — Supplementary Information. [file 41598_2023_34560_MOESM1_ESM.docx]

**Traumatic Brain Injury Related Death in Residents and Non-residents of 30 European countries: A Cross-sectional Study**

*Supplemental data*

**Figure S1: Crude and age-standardised TBI-related mortality rates per 100,000 person-years in 30 European countries in 2015 with a pooled age-standardised mortality rate; residents and non-resident combined, both sexes combined**

*St: standardised; CI: confidence interval; Cr: crude*

*Heterogeneity Assessment: I^2^ = 99.8% (95% CI: 99.8% to 99.8%)*

**Figure S2: Crude and age-standardised TBI-related mortality rates per 100,000 person-years in 30 European countries in 2015 with a pooled age-standardised mortality rate; residents and non-resident combined, males**

*St: standardised; CI: confidence interval; Cr: crude*

*Heterogeneity Assessment: I^2^ = 99.8% (95% CI: 99.8% to 99.8%)*

**Figure S3: Crude and age-standardised TBI-related mortality rates per 100,000 person-years in 30 European countries in 2015 with a pooled age-standardised mortality rate; residents and non-resident combined, females**

*St: standardised; CI: confidence interval; Cr: crude*

*Heterogeneity Assessment: I^2^ = 99.8% (95% CI: 99.8% to 99.8%)*

**Figure S4: Crude and age-standardised TBI-related mortality rates per 100,000 person-years in 30 European countries in 2015 with a pooled age-standardised mortality rate; residents, males**

*St: standardised; CI: confidence interval; Cr: crude*

*Heterogeneity Assessment: I^2^ = 99.8% (95% CI: 99.7% to 99.8%)*

**Figure S5: Crude and age-standardised TBI-related mortality rates per 100,000 person-years in 30 European countries in 2015 with a pooled age-standardised mortality rate; residents, females**

*St: standardised; CI: confidence interval; Cr: crude*

*Heterogeneity Assessment: I^2^ = 98.9% (95% CI: 98.7% to 99.0%)*

**Figure S6: Crude and age-standardised TBI-related mortality rates per 100,000 person-years in 30 European countries in 2015 with a pooled age-standardised mortality rate; non-residents, males**

*St: standardised; CI: confidence interval; Cr: crude*

*Heterogeneity Assessment: I^2^ = 97.2% (95% CI: 96.6% to 97.7%)*

**Figure S7: Crude and age-standardised TBI-related mortality rates per 100,000 person-years in 30 European countries in 2015 with a pooled age-standardised mortality rate; non-residents, females**

*St: standardised; CI: confidence interval; Cr: crude*

*Heterogeneity Assessment: I^2^ = 89.2% (95% CI: 85.7% to 91.8%)*

**Table S1: Distribution of TBI-related deaths in residents and non-residents of 30 European countries in 2015, stratified by country and sex**

| **Country** | **Both sexes** | | | | | **Males** | | | | | **Females** | | | | |
| --- | --- | --- | --- | --- | --- | --- | --- | --- | --- | --- | --- | --- | --- | --- | --- |
|  | **Residents** | | **Non-residents** | | **Total** | **Residents** | | **Non-residents** | | **Total** | **Residents** | | **Non-residents** | | **Total** |
|  | **Number** | **% Of all cases** | **Number** | **% Of all cases** |  | **Number** | **% Of all cases** | **Number** | **% Of all cases** |  | **Number** | **% Of all cases** | **Number** | **% Of all cases** |  |
| **AT** | 1005 | 94.99 | 53 | 5.01 | 1058 | 702 | 94.86 | 38 | 5.14 | 740 | 303 | 95.28 | 15 | 4.72 | 318 |
| **BE** | 1275 | 97.33 | 35 | 2.67 | 1310 | 733 | 96.45 | 27 | 3.55 | 760 | 542 | 98.55 | 8 | 1.45 | 550 |
| **BG** | 653 | 98.79 | 8 | 1.21 | 661 | 502 | 98.62 | 7 | 1.38 | 509 | 151 | 99.34 | 1 | 0.66 | 152 |
| **CY** | 53 | 92.98 | 4 | 7.02 | 57 | 37 | 92.50 | 3 | 7.50 | 40 | 16 | 94.12 | 1 | 5.88 | 17 |
| **CZ** | 1478 | 97.24 | 42 | 2.76 | 1520 | 1102 | 96.67 | 38 | 3.33 | 1140 | 376 | 98.95 | 4 | 1.05 | 380 |
| **DE** | 8048 | 98.72 | 104 | 1.28 | 8152 | 5117 | 98.31 | 88 | 1.69 | 5205 | 2931 | 99.46 | 16 | 0.54 | 2947 |
| **DK** | 359 | 98.63 | 5 | 1.37 | 364 | 238 | 98.76 | 3 | 1.24 | 241 | 121 | 98.37 | 2 | 1.63 | 123 |
| **EE** | 178 | 98.89 | 2 | 1.11 | 180 | 131 | 98.50 | 2 | 1.50 | 133 | 47 | 100.00 | 0 | 0.00 | 47 |
| **FI** | 1020 | 99.51 | 5 | 0.49 | 1025 | 678 | 99.27 | 5 | 0.73 | 683 | 342 | 100.00 | 0 | 0.00 | 342 |
| **GR** | 833 | 96.64 | 29 | 3.36 | 862 | 595 | 96.59 | 21 | 3.41 | 616 | 238 | 96.75 | 8 | 3.25 | 246 |
| **HR** | 512 | 96.97 | 16 | 3.03 | 528 | 365 | 96.82 | 12 | 3.18 | 377 | 147 | 97.35 | 4 | 2.65 | 151 |
| **HU** | 1250 | 97.81 | 28 | 2.19 | 1278 | 896 | 97.39 | 24 | 2.61 | 920 | 354 | 98.88 | 4 | 1.12 | 358 |
| **CH** | 827 | 95.50 | 39 | 4.50 | 866 | 511 | 94.45 | 30 | 5.55 | 541 | 316 | 97.23 | 9 | 2.77 | 325 |
| **IE** | 216 | 96.86 | 7 | 3.14 | 223 | 142 | 97.26 | 4 | 2.74 | 146 | 74 | 96.10 | 3 | 3.90 | 77 |
| **IS** | 16 | 100.00 | 0 | 0.00 | 16 | 8 | 100.00 | 0 | 0.00 | 8 | 8 | 100.00 | 0 | 0.00 | 8 |
| **IT** | 4792 | 97.34 | 131 | 2.66 | 4923 | 3164 | 96.91 | 101 | 3.09 | 3265 | 1628 | 98.19 | 30 | 1.81 | 1658 |
| **LT** | 526 | 99.62 | 2 | 0.38 | 528 | 393 | 99.49 | 2 | 0.51 | 395 | 133 | 100.00 | 0 | 0.00 | 133 |
| **LU** | 51 | 91.07 | 5 | 8.93 | 56 | 32 | 88.89 | 4 | 11.11 | 36 | 19 | 95.00 | 1 | 5.00 | 20 |
| **LV** | 321 | 99.69 | 1 | 0.31 | 322 | 263 | 99.62 | 1 | 0.38 | 264 | 58 | 100.00 | 0 | 0.00 | 58 |
| **MT** | 13 | 100.00 | 0 | 0.00 | 13 | 10 | 100.00 | 0 | 0.00 | 10 | 3 | 100.00 | 0 | 0.00 | 3 |
| **NL** | 1567 | 96.79 | 52 | 3.21 | 1619 | 931 | 96.58 | 33 | 3.42 | 964 | 636 | 97.25 | 18 | 2.75 | 654 |
| **NO** | 368 | 94.12 | 23 | 5.88 | 391 | 251 | 92.96 | 19 | 7.04 | 270 | 117 | 96.69 | 4 | 3.31 | 121 |
| **PT** | 1196 | 98.19 | 22 | 1.81 | 1218 | 824 | 98.56 | 12 | 1.44 | 836 | 372 | 97.38 | 10 | 2.62 | 382 |
| **RO** | 2086 | 99.62 | 8 | 0.38 | 2094 | 1648 | 99.88 | 2 | 0.12 | 1650 | 438 | 98.65 | 6 | 1.35 | 444 |
| **RS** | 785 | 100.00 | 0 | 0.00 | 785 | 595 | 100.00 | 0 | 0.00 | 595 | 190 | 100.00 | 0 | 0.00 | 190 |
| **SE** | 984 | 97.52 | 25 | 2.48 | 1009 | 675 | 99.26 | 5 | 0.74 | 680 | 309 | 100.00 | 0 | 0.00 | 309 |
| **SI** | 181 | 100.00 | 0 | 0.00 | 181 | 135 | 100.00 | 0 | 0.00 | 135 | 46 | 100.00 | 0 | 0.00 | 46 |
| **SK** | 783 | 98.86 | 9 | 1.14 | 792 | 621 | 99.04 | 6 | 0.96 | 627 | 162 | 98.18 | 3 | 1.82 | 165 |
| **TR** | 3284 | 89.17 | 399 | 10.83 | 3683 | 2581 | 88.57 | 333 | 11.43 | 2914 | 702 | 91.41 | 66 | 8.59 | 768 |
| **UK** | 4344 | 99.34 | 29 | 0.66 | 4373 | 2663 | 99.03 | 26 | 0.97 | 2689 | 1681 | 99.82 | 3 | 0.18 | 1684 |

*AT. Austria; BE. Belgium; BG. Bulgaria; CY. Cyprus; CZ. Czechia; DE. Germany; DK. Denmark; EE. Estonia; FI. Finland; GR. Greece; HR. Croatia; HU. Hungary; CH. Switzerland; IE. Ireland; IS. Island; IT. Italy; LT. Lithuania; LU. Luxemburg; LV. Latvia; MT. Malta; NL. Netherlands; NO. Norway; PT. Portugal; RO. Romania; RS. Serbia; SE. Sweden; SI. Slovenia; SK. Slovakia; TR. Turkey; UK. United Kingdom*

**Table S2: Distribution of TBI-related deaths in residents and non-residents of 30 European countries in 2015, stratified by country and age-group, both sexes combined**

| **Country** | **Residents** | | | | | | | | | | | | | **Non-residents** | | | | | | | | | | | | | **Grand total** |
| --- | --- | --- | --- | --- | --- | --- | --- | --- | --- | --- | --- | --- | --- | --- | --- | --- | --- | --- | --- | --- | --- | --- | --- | --- | --- | --- | --- |
|  | **Age group** | | | | | | | | | | | | **Total** | **Age group** | | | | | | | | | | | | **Total** |  |
|  | **0–4** | | **5–14** | | **15–34** | | **35–64** | | **65–84** | | **85+** | |  | **0–4** | | **5–14** | | **15–34** | | **35–64** | | **65–84** | | **85+** | |  |  |
|  | **N** | **%** | **N** | **%** | **N** | **%** | **N** | **%** | **N** | **%** | **N** | **%** |  | **N** | **%** | **N** | **%** | **N** | **%** | **N** | **%** | **N** | **%** | **N** | **%** |  |  |
| **AT** | 6 | 0.60 | 4 | 0.40 | 68 | 6.77 | 216 | 21.49 | 451 | 44.88 | 260 | 25.87 | 1005 | 1 | 1.89 | 1 | 1.89 | 12 | 22.64 | 20 | 37.74 | 14 | 26.42 | 5 | 9.43 | 53 | 1058 |
| **BE** | 8 | 0.63 | 4 | 0.31 | 139 | 10.90 | 362 | 28.39 | 459 | 36.00 | 303 | 23.76 | 1275 | 0 | 0.00 | 0 | 0.00 | 10 | 28.57 | 19 | 54.29 | 5 | 14.29 | 1 | 2.86 | 35 | 1310 |
| **BG** | 6 | 0.92 | 12 | 1.84 | 118 | 18.07 | 270 | 41.35 | 207 | 31.70 | 40 | 6.13 | 653 | 0 | 0.00 | 0 | 0.00 | 2 | 25.00 | 5 | 62.50 | 1 | 12.50 | 0 | 0.00 | 8 | 661 |
| **CY** | 1 | 1.89 | 0 | 0.00 | 14 | 26.42 | 11 | 20.75 | 18 | 33.96 | 9 | 16.98 | 53 | 0 | 0.00 | 0 | 0.00 | 2 | 50.00 | 2 | 50.00 | 0 | 0.00 | 0 | 0.00 | 4 | 57 |
| **CZ** | 9 | 0.61 | 14 | 0.95 | 193 | 13.06 | 557 | 37.69 | 523 | 35.39 | 182 | 12.31 | 1478 | 0 | 0.00 | 0 | 0.00 | 18 | 42.86 | 23 | 54.76 | 0 | 0.00 | 1 | 2.38 | 42 | 1520 |
| **DE** | 34 | 0.42 | 32 | 0.40 | 601 | 7.47 | 1690 | 21.00 | 3487 | 43.33 | 2204 | 27.39 | 8048 | 0 | 0.00 | 0 | 0.00 | 22 | 21.15 | 66 | 63.46 | 12 | 11.54 | 4 | 3.85 | 104 | 8152 |
| **DK** | 3 | 0.84 | 3 | 0.84 | 68 | 18.94 | 113 | 31.48 | 102 | 28.41 | 70 | 19.50 | 359 | 0 | 0.00 | 0 | 0.00 | 1 | 20.00 | 4 | 80.00 | 0 | 0.00 | 0 | 0.00 | 5 | 364 |
| **EE** | 1 | 0.56 | 4 | 2.25 | 24 | 13.48 | 91 | 51.12 | 49 | 27.53 | 9 | 5.06 | 178 | 0 | 0.00 | 0 | 0.00 | 0 | 0.00 | 1 | 50.00 | 1 | 50.00 | 0 | 0.00 | 2 | 180 |
| **FI** | 1 | 0.10 | 13 | 1.27 | 77 | 7.55 | 281 | 27.55 | 396 | 38.82 | 252 | 24.71 | 1020 | 0 | 0.00 | 0 | 0.00 | 2 | 40.00 | 3 | 60.00 | 0 | 0.00 | 0 | 0.00 | 5 | 1025 |
| **GR** | 4 | 0.48 | 5 | 0.60 | 142 | 17.05 | 211 | 25.33 | 323 | 38.78 | 148 | 17.77 | 833 | 0 | 0.00 | 2 | 6.90 | 7 | 24.14 | 13 | 44.83 | 6 | 20.69 | 1 | 3.45 | 29 | 862 |
| **HR** | 1 | 0.20 | 7 | 1.37 | 47 | 9.18 | 156 | 30.47 | 228 | 44.53 | 73 | 14.26 | 512 | 0 | 0.00 | 0 | 0.00 | 0 | 0.00 | 6 | 37.50 | 9 | 56.25 | 1 | 6.25 | 16 | 528 |
| **HU** | 9 | 0.72 | 6 | 0.48 | 125 | 10.00 | 463 | 37.04 | 480 | 38.40 | 167 | 13.36 | 1250 | 0 | 0.00 | 1 | 3.57 | 6 | 21.43 | 17 | 60.71 | 4 | 14.29 | 0 | 0.00 | 28 | 1278 |
| **CH** | 6 | 0.73 | 3 | 0.36 | 48 | 5.80 | 134 | 16.20 | 329 | 39.78 | 307 | 37.12 | 827 | 0 | 0.00 | 0 | 0.00 | 8 | 20.51 | 18 | 46.15 | 9 | 23.08 | 4 | 10.26 | 39 | 866 |
| **IE** | 4 | 1.85 | 1 | 0.46 | 34 | 15.74 | 73 | 33.80 | 71 | 32.87 | 33 | 15.28 | 216 | 0 | 0.00 | 0 | 0.00 | 1 | 14.29 | 2 | 28.57 | 4 | 57.14 | 0 | 0.00 | 7 | 223 |
| **IS** | 1 | 6.25 | 0 | 0.00 | 5 | 31.25 | 1 | 6.25 | 5 | 31.25 | 4 | 25.00 | 16 | 0 | 0 | 0 | 0 | 0 | 0.00 | 0 | 0 | 0 | 0 | 0 | 0 | 0 | 16 |
| **IT** | 13 | 0.27 | 28 | 0.58 | 449 | 9.37 | 1089 | 22.73 | 1946 | 40.61 | 1267 | 26.44 | 4792 | 3 | 2.29 | 0 | 0.00 | 45 | 34.35 | 63 | 48.09 | 14 | 10.69 | 6 | 4.58 | 131 | 4923 |
| **LT** | 3 | 0.57 | 1 | 0.19 | 53 | 10.08 | 284 | 53.99 | 145 | 27.57 | 40 | 7.60 | 526 | 0 | 0.00 | 0 | 0.00 | 0 | 0.00 | 2 | 100.00 | 0 | 0.00 | 0 | 0.00 | 2 | 528 |
| **LU** | 1 | 1.96 | 0 | 0.00 | 5 | 9.80 | 16 | 31.37 | 19 | 37.25 | 10 | 19.61 | 51 | 1 | 20.00 | 0 | 0.00 | 1 | 20.00 | 3 | 60.00 | 0 | 0.00 | 0 | 0.00 | 5 | 56 |
| **LV** | 5 | 1.56 | 3 | 0.93 | 41 | 12.77 | 172 | 53.58 | 82 | 25.55 | 18 | 5.61 | 321 | 0 | 0.00 | 0 | 0.00 | 0 | 0.00 | 1 | 100.00 | 0 | 0.00 | 0 | 0.00 | 1 | 322 |
| **MT** | 0 | 0.00 | 1 | 7.69 | 3 | 23.08 | 3 | 23.08 | 6 | 46.15 | 0 | 0.00 | 13 | 0 | 0 | 0 | 0 | 0 | 0.00 | 0 | 0 | 0 | 0 | 0 | 0 | 0 | 13 |
| **NL** | 12 | 0.77 | 16 | 1.02 | 153 | 9.76 | 336 | 21.44 | 591 | 37.72 | 459 | 29.29 | 1567 | 1 | 1.92 | 1 | 1.92 | 19 | 36.54 | 22 | 42.31 | 7 | 13.46 | 2 | 3.85 | 52 | 1619 |
| **NO** | 0 | 0.00 | 2 | 0.54 | 36 | 9.78 | 97 | 26.36 | 116 | 31.52 | 117 | 31.79 | 368 | 0 | 0.00 | 1 | 4.35 | 9 | 39.13 | 12 | 52.17 | 1 | 4.35 | 0 | 0.00 | 23 | 391 |
| **PT** | 5 | 0.42 | 8 | 0.67 | 100 | 8.36 | 371 | 31.02 | 463 | 38.71 | 249 | 20.82 | 1196 | 0 | 0.00 | 0 | 0.00 | 4 | 18.18 | 7 | 31.82 | 6 | 27.27 | 5 | 22.73 | 22 | 1218 |
| **RO** | 36 | 1.73 | 31 | 1.49 | 301 | 14.43 | 994 | 47.65 | 635 | 30.44 | 89 | 4.27 | 2086 | 0 | 0.00 | 0 | 0.00 | 3 | 37.50 | 3 | 37.50 | 2 | 25.00 | 0 | 0.00 | 8 | 2094 |
| **RS** | 8 | 1.02 | 5 | 0.64 | 100 | 12.74 | 300 | 38.22 | 311 | 39.62 | 61 | 7.77 | 785 | 0 | 0 | 0 | 0 | 0 | 0.00 | 0 | 0 | 0 | 0 | 0 | 0 | 0 | 785 |
| **SE** | 3 | 0.30 | 1 | 0.10 | 107 | 10.87 | 168 | 17.07 | 386 | 39.23 | 319 | 32.42 | 984 | 1 | 4.00 | 0 | 0.00 | 6 | 24.00 | 13 | 52.00 | 4 | 16.00 | 1 | 4.00 | 25 | 1009 |
| **SI** | 0 | 0.00 | 1 | 0.55 | 9 | 4.97 | 45 | 24.86 | 89 | 49.17 | 37 | 20.44 | 181 | 0 | 0 | 0 | 0 | 0 | 0.00 | 0 | 0 | 0 | 0 | 0 | 0 | 0 | 181 |
| **SK** | 4 | 0.51 | 6 | 0.77 | 86 | 10.98 | 373 | 47.64 | 252 | 32.18 | 62 | 7.92 | 783 | 0 | 0.00 | 0 | 0.00 | 3 | 33.33 | 4 | 44.44 | 1 | 11.11 | 1 | 11.11 | 9 | 792 |
| **TR** | 161 | 4.90 | 152 | 4.63 | 885 | 26.95 | 1246 | 37.94 | 701 | 21.35 | 139 | 4.23 | 3284 | 20 | 5.01 | 48 | 12.03 | 228 | 57.14 | 86 | 21.55 | 16 | 4.01 | 1 | 0.25 | 399 | 3683 |
| **UK** | 25 | 0.58 | 27 | 0.62 | 346 | 7.97 | 884 | 20.35 | 1678 | 38.63 | 1384 | 31.86 | 4344 | 1 | 3.45 | 0 | 0.00 | 10 | 34.48 | 10 | 34.48 | 7 | 24.14 | 1 | 3.45 | 29 | 4373 |
| **TOTAL** | 370 | 0.95 | 390 | 1.00 | 4377 | 11.22 | 11007 | 28.22 | 14548 | 37.30 | 8312 | 21.31 | 39004 | 28 | 2.59 | 54 | 4.99 | 419 | 38.69 | 425 | 39.24 | 123 | 11.36 | 34 | 3.14 | 1083 | 40087 |

*AT. Austria; BE. Belgium; BG. Bulgaria; CY. Cyprus; CZ. Czechia; DE. Germany; DK. Denmark; EE. Estonia; FI. Finland; GR. Greece; HR. Croatia; HU. Hungary; CH. Switzerland; IE. Ireland; IS. Island; IT. Italy; LT. Lithuania; LU. Luxemburg; LV. Latvia; MT. Malta; NL. Netherlands; NO. Norway; PT. Portugal; RO. Romania; RS. Serbia; SE. Sweden; SI. Slovenia; SK. Slovakia; TR. Turkey; UK. United Kingdom*

**Table S3: Distribution of TBI-related deaths in residents and non-residents of 30 European countries in 2015, stratified by country and age group, males**

| **Country** | **Residents** | | | | | | | | | | | | | **Non-residents** | | | | | | | | | | | | | **Grand total** |
| --- | --- | --- | --- | --- | --- | --- | --- | --- | --- | --- | --- | --- | --- | --- | --- | --- | --- | --- | --- | --- | --- | --- | --- | --- | --- | --- | --- |
|  | **Age group** | | | | | | | | | | | | **Total** | **Age group** | | | | | | | | | | | | **Total** |  |
|  | **0–4** | | **5–14** | | **15–34** | | **35–64** | | **65–84** | | **85+** | |  | **0–4** | | **5–14** | | **15–34** | | **35–64** | | **65–84** | | **85+** | |  |  |
|  | **N** | **%** | **N** | **%** | **N** | **%** | **N** | **%** | **N** | **%** | **N** | **%** |  | **N** | **%** | **N** | **%** | **N** | **%** | **N** | **%** | **N** | **%** | **N** | **%** |  |  |
| **AT** | 4 | 0.57 | 2 | 0.28 | 59 | 8.40 | 183 | 26.07 | 333 | 47.44 | 121 | 17.24 | 702 | 0 | 0.00 | 0 | 0.00 | 8 | 21.05 | 16 | 42.11 | 11 | 28.95 | 3 | 7.89 | 38 | 740 |
| **BE** | 6 | 0.82 | 2 | 0.27 | 113 | 15.42 | 246 | 33.56 | 245 | 33.42 | 121 | 16.51 | 733 | 0 | 0.00 | 0 | 0.00 | 8 | 29.63 | 15 | 55.56 | 4 | 14.81 | 0 | 0.00 | 27 | 760 |
| **BG** | 4 | 0.80 | 10 | 1.99 | 98 | 19.52 | 230 | 45.82 | 135 | 26.89 | 25 | 4.98 | 502 | 0 | 0.00 | 0 | 0.00 | 2 | 28.57 | 5 | 71.43 | 0 | 0.00 | 0 | 0.00 | 7 | 509 |
| **CY** | 0 | 0.00 | 0 | 0.00 | 14 | 37.84 | 8 | 21.62 | 10 | 27.03 | 5 | 13.51 | 37 | 0 | 0.00 | 0 | 0.00 | 1 | 33.33 | 2 | 66.67 | 0 | 0.00 | 0 | 0.00 | 3 | 40 |
| **CZ** | 5 | 0.45 | 6 | 0.54 | 157 | 14.25 | 467 | 42.38 | 379 | 34.39 | 88 | 7.99 | 1102 | 0 | 0.00 | 0 | 0.00 | 16 | 42.11 | 21 | 55.26 | 0 | 0.00 | 1 | 2.63 | 38 | 1140 |
| **DE** | 24 | 0.47 | 25 | 0.49 | 483 | 9.44 | 1369 | 26.75 | 2262 | 44.21 | 954 | 18.64 | 5117 | 0 | 0.00 | 0 | 0.00 | 20 | 22.73 | 57 | 64.77 | 7 | 7.95 | 4 | 4.55 | 88 | 5205 |
| **DK** | 2 | 0.84 | 2 | 0.84 | 58 | 24.37 | 82 | 34.45 | 64 | 26.89 | 30 | 12.61 | 238 | 0 | 0.00 | 0 | 0.00 | 1 | 33.33 | 2 | 66.67 | 0 | 0.00 | 0 | 0.00 | 3 | 241 |
| **EE** | 0 | 0.00 | 3 | 2.29 | 17 | 12.98 | 73 | 55.73 | 33 | 25.19 | 5 | 3.82 | 131 | 0 | 0.00 | 0 | 0.00 | 0 | 0.00 | 1 | 50.00 | 1 | 50.00 | 0 | 0.00 | 2 | 133 |
| **FI** | 1 | 0.15 | 7 | 1.03 | 63 | 9.29 | 235 | 34.66 | 269 | 39.68 | 103 | 15.19 | 678 | 0 | 0.00 | 0 | 0.00 | 2 | 40.00 | 3 | 60.00 | 0 | 0.00 | 0 | 0.00 | 5 | 683 |
| **GR** | 2 | 0.34 | 2 | 0.34 | 116 | 19.50 | 180 | 30.25 | 214 | 35.97 | 81 | 13.61 | 595 | 0 | 0.00 | 0 | 0.00 | 4 | 19.05 | 11 | 52.38 | 6 | 28.57 | 0 | 0.00 | 21 | 616 |
| **HR** | 1 | 0.27 | 4 | 1.10 | 41 | 11.23 | 135 | 36.99 | 151 | 41.37 | 33 | 9.04 | 365 | 0 | 0.00 | 0 | 0.00 | 0 | 0.00 | 5 | 41.67 | 7 | 58.33 | 0 | 0.00 | 12 | 377 |
| **HU** | 5 | 0.56 | 4 | 0.45 | 97 | 10.83 | 400 | 44.64 | 319 | 35.60 | 71 | 7.92 | 896 | 0 | 0.00 | 0 | 0.00 | 5 | 20.83 | 15 | 62.50 | 4 | 16.67 | 0 | 0.00 | 24 | 920 |
| **CH** | 5 | 0.98 | 0 | 0.00 | 43 | 8.41 | 94 | 18.40 | 225 | 44.03 | 144 | 28.18 | 511 | 0 | 0.00 | 0 | 0.00 | 7 | 23.33 | 14 | 46.67 | 8 | 26.67 | 1 | 3.33 | 30 | 541 |
| **IE** | 2 | 1.41 | 1 | 0.70 | 29 | 20.42 | 58 | 40.85 | 37 | 26.06 | 15 | 10.56 | 142 | 0 | 0.00 | 0 | 0.00 | 0 | 0.00 | 1 | 25.00 | 3 | 75.00 | 0 | 0.00 | 4 | 146 |
| **IS** | 0 | 0.00 | 0 | 0.00 | 4 | 50.00 | 1 | 12.50 | 3 | 37.50 | 0 | 0.00 | 8 | 0 | 0.00 | 0 | 0.00 | 0 | 0.00 | 0 | 0.00 | 0 | 0.00 | 0 | 0.00 | 0 | 8 |
| **IT** | 8 | 0.25 | 18 | 0.57 | 371 | 11.73 | 887 | 28.03 | 1284 | 40.58 | 596 | 18.84 | 3164 | 2 | 1.98 | 0 | 0.00 | 34 | 33.66 | 53 | 52.48 | 8 | 7.92 | 4 | 3.96 | 101 | 3265 |
| **LT** | 2 | 0.51 | 0 | 0.00 | 42 | 10.69 | 236 | 60.05 | 96 | 24.43 | 17 | 4.33 | 393 | 0 | 0.00 | 0 | 0.00 | 0 | 0.00 | 2 | 100.00 | 0 | 0.00 | 0 | 0.00 | 2 | 395 |
| **LU** | 1 | 3.13 | 0 | 0.00 | 5 | 15.63 | 12 | 37.50 | 9 | 28.13 | 5 | 15.63 | 32 | 1 | 25.00 | 0 | 0.00 | 1 | 25.00 | 2 | 50.00 | 0 | 0.00 | 0 | 0.00 | 4 | 36 |
| **LV** | 4 | 1.52 | 2 | 0.76 | 35 | 13.31 | 155 | 58.94 | 59 | 22.43 | 8 | 3.04 | 263 | 0 | 0.00 | 0 | 0.00 | 0 | 0.00 | 1 | 100.00 | 0 | 0.00 | 0 | 0.00 | 1 | 264 |
| **MT** | 0 | 0.00 | 1 | 10.00 | 2 | 20.00 | 3 | 30.00 | 4 | 40.00 | 0 | 0.00 | 10 | 0 | 0.00 | 0 | 0.00 | 0 | 0.00 | 0 | 0.00 | 0 | 0.00 | 0 | 0.00 | 0 | 10 |
| **NL** | 8 | 0.86 | 9 | 0.97 | 124 | 13.32 | 243 | 26.10 | 350 | 37.59 | 197 | 21.16 | 931 | 0 | 0.00 | 1 | 3.03 | 13 | 39.39 | 17 | 51.52 | 2 | 6.06 | 0 | 0.00 | 33 | 964 |
| **NO** | 0 | 0.00 | 2 | 0.80 | 30 | 11.95 | 81 | 32.27 | 82 | 32.67 | 56 | 22.31 | 251 | 0 | 0.00 | 0 | 0.00 | 8 | 42.11 | 11 | 57.89 | 0 | 0.00 | 0 | 0.00 | 19 | 270 |
| **PT** | 3 | 0.36 | 6 | 0.73 | 79 | 9.59 | 275 | 33.37 | 315 | 38.23 | 146 | 17.72 | 824 | 0 | 0.00 | 0 | 0.00 | 4 | 33.33 | 5 | 41.67 | 2 | 16.67 | 1 | 8.33 | 12 | 836 |
| **RO** | 23 | 1.40 | 21 | 1.27 | 254 | 15.41 | 848 | 51.46 | 453 | 27.49 | 49 | 2.97 | 1648 | 0 | 0.00 | 0 | 0.00 | 1 | 50.00 | 1 | 50.00 | 0 | 0.00 | 0 | 0.00 | 2 | 1650 |
| **RS** | 6 | 1.01 | 4 | 0.67 | 81 | 13.61 | 255 | 42.86 | 214 | 35.97 | 35 | 5.88 | 595 | 0 | 0.00 | 0 | 0.00 | 0 | 0.00 | 0 | 0.00 | 0 | 0.00 | 0 | 0.00 | 0 | 595 |
| **SE** | 3 | 0.44 | 1 | 0.15 | 77 | 11.41 | 142 | 21.04 | 274 | 40.59 | 178 | 26.37 | 675 | 0 | 0.00 | 0 | 0.00 | 1 | 20.00 | 2 | 40.00 | 2 | 40.00 | 0 | 0.00 | 5 | 680 |
| **SI** | 0 | 0.00 | 0 | 0.00 | 8 | 5.93 | 42 | 31.11 | 62 | 45.93 | 23 | 17.04 | 135 | 0 | 0.00 | 0 | 0.00 | 0 | 0.00 | 0 | 0.00 | 0 | 0.00 | 0 | 0.00 | 0 | 135 |
| **SK** | 3 | 0.48 | 4 | 0.64 | 72 | 11.59 | 335 | 53.95 | 175 | 28.18 | 32 | 5.15 | 621 | 0 | 0.00 | 0 | 0.00 | 2 | 33.33 | 2 | 33.33 | 1 | 16.67 | 1 | 16.67 | 6 | 627 |
| **TR** | 98 | 3.80 | 106 | 4.11 | 721 | 27.93 | 1035 | 40.10 | 538 | 20.84 | 83 | 3.22 | 2581 | 10 | 3.00 | 36 | 10.81 | 207 | 62.16 | 70 | 21.02 | 9 | 2.70 | 1 | 0.30 | 333 | 2914 |
| **UK** | 13 | 0.49 | 16 | 0.60 | 284 | 10.66 | 690 | 25.91 | 1012 | 38.00 | 648 | 24.33 | 2663 | 0 | 0.00 | 0 | 0.00 | 10 | 38.46 | 10 | 38.46 | 6 | 23.08 | 0 | 0.00 | 26 | 2689 |
| **TOTAL** | 233 | 0.88 | 258 | 0.97 | 3577 | 13.48 | 9000 | 33.91 | 9606 | 36.19 | 3869 | 14.58 | 26543 | 13 | 1.54 | 37 | 4.37 | 355 | 41.96 | 344 | 40.66 | 81 | 9.57 | 16 | 1.89 | 846 | 27389 |

*AT. Austria; BE. Belgium; BG. Bulgaria; CY. Cyprus; CZ. Czechia; DE. Germany; DK. Denmark; EE. Estonia; FI. Finland; GR. Greece; HR. Croatia; HU. Hungary; CH. Switzerland; IE. Ireland; IS. Island; IT. Italy; LT. Lithuania; LU. Luxemburg; LV. Latvia; MT. Malta; NL. Netherlands; NO. Norway; PT. Portugal; RO. Romania; RS. Serbia; SE. Sweden; SI. Slovenia; SK. Slovakia; TR. Turkey; UK. United Kingdom*

**Table S4: Distribution of TBI-related deaths in residents and non-residents of 30 European countries in 2015, stratified by country and age-group, females**

| **Country** | **Residents** | | | | | | | | | | | | | **Non-residents** | | | | | | | | | | | | | **Grand total** |
| --- | --- | --- | --- | --- | --- | --- | --- | --- | --- | --- | --- | --- | --- | --- | --- | --- | --- | --- | --- | --- | --- | --- | --- | --- | --- | --- | --- |
|  | **Age group** | | | | | | | | | | | | **Total** | **Age group** | | | | | | | | | | | | **Total** |  |
|  | **0–4** | | **5–14** | | **15–34** | | **35–64** | | **65–84** | | **85+** | |  | **0–4** | | **5–14** | | **15–34** | | **35–64** | | **65–84** | | **85+** | |  |  |
|  | **N** | **%** | **N** | **%** | **N** | **%** | **N** | **%** | **N** | **%** | **N** | **%** |  | **N** | **%** | **N** | **%** | **N** | **%** | **N** | **%** | **N** | **%** | **N** | **%** |  |  |
| **AT** | 2 | 0.66 | 2 | 0.66 | 9 | 2.97 | 33 | 10.89 | 118 | 38.94 | 139 | 45.87 | 303 | 1 | 6.67 | 1 | 6.67 | 4 | 26.67 | 4 | 26.67 | 3 | 20.00 | 2 | 13.33 | 15 | 318 |
| **BE** | 2 | 0.37 | 2 | 0.37 | 26 | 4.80 | 116 | 21.40 | 214 | 39.48 | 182 | 33.58 | 542 | 0 | 0.00 | 0 | 0.00 | 2 | 25.00 | 4 | 50.00 | 1 | 12.50 | 1 | 12.50 | 8 | 550 |
| **BG** | 2 | 1.32 | 2 | 1.32 | 20 | 13.25 | 40 | 26.49 | 72 | 47.68 | 15 | 9.93 | 151 | 0 | 0.00 | 0 | 0.00 | 0 | 0.00 | 0 | 0.00 | 1 | 100.00 | 0 | 0.00 | 1 | 152 |
| **CY** | 1 | 6.25 | 0 | 0.00 | 0 | 0.00 | 3 | 18.75 | 8 | 50.00 | 4 | 25.00 | 16 | 0 | 0.00 | 0 | 0.00 | 1 | 100.00 | 0 | 0.00 | 0 | 0.00 | 0 | 0.00 | 1 | 17 |
| **CZ** | 4 | 1.06 | 8 | 2.13 | 36 | 9.57 | 90 | 23.94 | 144 | 38.30 | 94 | 25.00 | 376 | 0 | 0.00 | 0 | 0.00 | 2 | 50.00 | 2 | 50.00 | 0 | 0.00 | 0 | 0.00 | 4 | 380 |
| **DE** | 10 | 0.34 | 7 | 0.24 | 118 | 4.03 | 321 | 10.95 | 1225 | 41.79 | 1250 | 42.65 | 2931 | 0 | 0.00 | 0 | 0.00 | 2 | 12.50 | 9 | 56.25 | 5 | 31.25 | 0 | 0.00 | 16 | 2947 |
| **DK** | 1 | 0.83 | 1 | 0.83 | 10 | 8.26 | 31 | 25.62 | 38 | 31.40 | 40 | 33.06 | 121 | 0 | 0.00 | 0 | 0.00 | 0 | 0.00 | 2 | 100.00 | 0 | 0.00 | 0 | 0.00 | 2 | 123 |
| **EE** | 1 | 2.13 | 1 | 2.13 | 7 | 14.89 | 18 | 38.30 | 16 | 34.04 | 4 | 8.51 | 47 | 0 | 0.00 | 0 | 0.00 | 0 | 0.00 | 0 | 0.00 | 0 | 0.00 | 0 | 0.00 | 0 | 47 |
| **FI** | 0 | 0.00 | 6 | 1.75 | 14 | 4.09 | 46 | 13.45 | 127 | 37.13 | 149 | 43.57 | 342 | 0 | 0.00 | 0 | 0.00 | 0 | 0.00 | 0 | 0.00 | 0 | 0.00 | 0 | 0.00 | 0 | 342 |
| **GR** | 2 | 0.84 | 3 | 1.26 | 26 | 10.92 | 31 | 13.03 | 109 | 45.80 | 67 | 28.15 | 238 | 0 | 0.00 | 2 | 25.00 | 3 | 37.50 | 2 | 25.00 | 0 | 0.00 | 1 | 12.50 | 8 | 246 |
| **HR** | 0 | 0.00 | 3 | 2.04 | 6 | 4.08 | 21 | 14.29 | 77 | 52.38 | 40 | 27.21 | 147 | 0 | 0.00 | 0 | 0.00 | 0 | 0.00 | 1 | 25.00 | 2 | 50.00 | 1 | 25.00 | 4 | 151 |
| **HU** | 4 | 1.13 | 2 | 0.56 | 28 | 7.91 | 63 | 17.80 | 161 | 45.48 | 96 | 27.12 | 354 | 0 | 0.00 | 1 | 25.00 | 1 | 25.00 | 2 | 50.00 | 0 | 0.00 | 0 | 0.00 | 4 | 358 |
| **CH** | 1 | 0.32 | 3 | 0.95 | 5 | 1.58 | 40 | 12.66 | 104 | 32.91 | 163 | 51.58 | 316 | 0 | 0.00 | 0 | 0.00 | 1 | 11.11 | 4 | 44.44 | 1 | 11.11 | 3 | 33.33 | 9 | 325 |
| **IE** | 2 | 2.70 | 0 | 0.00 | 5 | 6.76 | 15 | 20.27 | 34 | 45.95 | 18 | 24.32 | 74 | 0 | 0.00 | 0 | 0.00 | 1 | 33.33 | 1 | 33.33 | 1 | 33.33 | 0 | 0.00 | 3 | 77 |
| **IS** | 1 | 12.50 | 0 | 0.00 | 1 | 12.50 | 0 | 0.00 | 2 | 25.00 | 4 | 50.00 | 8 | 0 | 0.00 | 0 | 0.00 | 0 | 0.00 | 0 | 0.00 | 0 | 0.00 | 0 | 0.00 | 0 | 8 |
| **IT** | 5 | 0.31 | 10 | 0.61 | 78 | 4.79 | 202 | 12.41 | 662 | 40.66 | 671 | 41.22 | 1628 | 1 | 3.33 | 0 | 0.00 | 11 | 36.67 | 10 | 33.33 | 6 | 20.00 | 2 | 6.67 | 30 | 1658 |
| **LT** | 1 | 0.75 | 1 | 0.75 | 11 | 8.27 | 48 | 36.09 | 49 | 36.84 | 23 | 17.29 | 133 | 0 | 0.00 | 0 | 0.00 | 0 | 0.00 | 0 | 0.00 | 0 | 0.00 | 0 | 0.00 | 0 | 133 |
| **LU** | 0 | 0.00 | 0 | 0.00 | 0 | 0.00 | 4 | 21.05 | 10 | 52.63 | 5 | 26.32 | 19 | 0 | 0.00 | 0 | 0.00 | 0 | 0.00 | 1 | 100.00 | 0 | 0.00 | 0 | 0.00 | 1 | 20 |
| **LV** | 1 | 1.72 | 1 | 1.72 | 6 | 10.34 | 17 | 29.31 | 23 | 39.66 | 10 | 17.24 | 58 | 0 | 0.00 | 0 | 0.00 | 0 | 0.00 | 0 | 0.00 | 0 | 0.00 | 0 | 0.00 | 0 | 58 |
| **MT** | 0 | 0.00 | 0 | 0.00 | 1 | 33.33 | 0 | 0.00 | 2 | 66.67 | 0 | 0.00 | 3 | 0 | 0.00 | 0 | 0.00 | 0 | 0.00 | 0 | 0.00 | 0 | 0.00 | 0 | 0.00 | 0 | 3 |
| **NL** | 4 | 0.63 | 7 | 1.10 | 29 | 4.56 | 93 | 14.62 | 241 | 37.89 | 262 | 41.19 | 636 | 1 | 5.56 | 0 | 0.00 | 6 | 33.33 | 4 | 22.22 | 5 | 27.78 | 2 | 11.11 | 18 | 654 |
| **NO** | 0 | 0.00 | 0 | 0.00 | 6 | 5.13 | 16 | 13.68 | 34 | 29.06 | 61 | 52.14 | 117 | 0 | 0.00 | 1 | 25.00 | 1 | 25.00 | 1 | 25.00 | 1 | 25.00 | 0 | 0.00 | 4 | 121 |
| **PT** | 2 | 0.54 | 2 | 0.54 | 21 | 5.65 | 96 | 25.81 | 148 | 39.78 | 103 | 27.69 | 372 | 0 | 0.00 | 0 | 0.00 | 0 | 0.00 | 2 | 20.00 | 4 | 40.00 | 4 | 40.00 | 10 | 382 |
| **RO** | 13 | 2.97 | 10 | 2.28 | 47 | 10.73 | 146 | 33.33 | 182 | 41.55 | 40 | 9.13 | 438 | 0 | 0.00 | 0 | 0.00 | 2 | 33.33 | 2 | 33.33 | 2 | 33.33 | 0 | 0.00 | 6 | 444 |
| **RS** | 2 | 1.05 | 1 | 0.53 | 19 | 10.00 | 45 | 23.68 | 97 | 51.05 | 26 | 13.68 | 190 | 0 | 0.00 | 0 | 0.00 | 0 | 0.00 | 0 | 0.00 | 0 | 0.00 | 0 | 0.00 | 0 | 190 |
| **SE** | 0 | 0.00 | 0 | 0.00 | 30 | 9.71 | 26 | 8.41 | 112 | 36.25 | 141 | 45.63 | 309 | 0 | 0.00 | 0 | 0.00 | 0 | 0.00 | 0 | 0.00 | 0 | 0.00 | 0 | 0.00 | 0 | 309 |
| **SI** | 0 | 0.00 | 1 | 2.17 | 1 | 2.17 | 3 | 6.52 | 27 | 58.70 | 14 | 30.43 | 46 | 0 | 0.00 | 0 | 0.00 | 0 | 0.00 | 0 | 0.00 | 0 | 0.00 | 0 | 0.00 | 0 | 46 |
| **SK** | 1 | 0.62 | 2 | 1.23 | 14 | 8.64 | 38 | 23.46 | 77 | 47.53 | 30 | 18.52 | 162 | 0 | 0.00 | 0 | 0.00 | 1 | 33.33 | 2 | 66.67 | 0 | 0.00 | 0 | 0.00 | 3 | 165 |
| **TR** | 63 | 8.97 | 46 | 6.55 | 164 | 23.36 | 210 | 29.91 | 163 | 23.22 | 56 | 7.98 | 702 | 10 | 15.15 | 12 | 18.18 | 21 | 31.82 | 16 | 24.24 | 7 | 10.61 | 0 | 0.00 | 66 | 768 |
| **UK** | 12 | 0.71 | 11 | 0.65 | 62 | 3.69 | 194 | 11.54 | 666 | 39.62 | 736 | 43.78 | 1681 | 1 | 33.33 | 0 | 0.00 | 0 | 0.00 | 0 | 0.00 | 1 | 33.33 | 1 | 33.33 | 3 | 1684 |
| **TOTAL** | 137 | 1.10 | 132 | 1.06 | 800 | 6.42 | 2006 | 16.099518 | 4942 | 39.66 | 4443 | 35.66 | 12460 | 14 | 6.48 | 17 | 7.87 | 59 | 27.31 | 69 | 31.94 | 40 | 18.52 | 17 | 7.87 | 216 | 12676 |

*AT. Austria; BE. Belgium; BG. Bulgaria; CY. Cyprus; CZ. Czechia; DE. Germany; DK. Denmark; EE. Estonia; FI. Finland; GR. Greece; HR. Croatia; HU. Hungary; CH. Switzerland; IE. Ireland; IS. Island; IT. Italy; LT. Lithuania; LU. Luxemburg; LV. Latvia; MT. Malta; NL. Netherlands; NO. Norway; PT. Portugal; RO. Romania; RS. Serbia; SE. Sweden; SI. Slovenia; SK. Slovakia; TR. Turkey; UK. United Kingdom*

**Table S5: Overall injury-related deaths and TBI-related deaths in 30 countries of Europe in 2015 by cause of injury**

| **Cause of Injury** | **Both sexes** | | **Males** | | **Females** | |
| --- | --- | --- | --- | --- | --- | --- |
|  | **Number** | **% Of all cases** | **Number** | **% Of all cases** | **Number** | **% Of all cases** |
|  | **All injury-related deaths** | | | | | |
| **Falls** | 70,011 | 22.94 | 34,920 | 18.13 | 35,084 | 31.20 |
| **Suicide** | 69,491 | 22.77 | 53,171 | 27.61 | 16,301 | 14.50 |
| **Violence** | 5128 | 1.68 | 3416 | 1.77 | 1709 | 1.52 |
| **Traffic** | 40,834 | 13.38 | 31,058 | 16.13 | 9747 | 8.67 |
| **Other** | 119,677 | 39.22 | 70,027 | 36.36 | 49,611 | 44.12 |
| **TOTAL** | 305,141 | 100.00 | 192,592 | 100.00 | 112,452 | 100.00 |
| **TBI-related deaths (residents and non-residents combined)** | | | | | | |
| **Falls** | 18,705 | 46.66 | 11,188 | 40.85 | 7511 | 59.25 |
| **Suicide** | 3827 | 9.55 | 3307 | 12.07 | 518 | 4.09 |
| **Violence** | 983 | 2.45 | 686 | 2.50 | 294 | 2.32 |
| **Traffic** | 8838 | 22.05 | 6748 | 24.64 | 2079 | 16.40 |
| **Other** | 7734 | 19.29 | 5460 | 19.94 | 2274 | 17.94 |
| **TOTAL** | 40,087 | 100.00 | 27,389 | 100.00 | 12,676 | 100.00 |
| **TBI-related deaths among residents** | | | | | | |
| **Falls** | 18,479 | 47.38 | 11,028 | 41.55 | 7451 | 59.80 |
| **Suicide** | 3783 | 9.70 | 3275 | 12.34 | 508 | 4.08 |
| **Violence** | 933 | 2.39 | 641 | 2.41 | 292 | 2.34 |
| **Traffic** | 8450 | 21.66 | 6468 | 24.37 | 1981 | 15.90 |
| **Other** | 7359 | 18.87 | 5131 | 19.33 | 2228 | 17.88 |
| **TOTAL** | 39,004 | 100.00 | 26,543 | 100.00 | 12,460 | 100.00 |
| **TBI-related deaths among non-residents** | | | | | | |
| **Falls** | 226 | 20.87 | 160 | 18.91 | 60 | 27.78 |
| **Suicide** | 44 | 4.06 | 32 | 3.78 | 10 | 4.63 |
| **Violence** | 50 | 4.62 | 45 | 5.32 | 2 | 0.93 |
| **Traffic** | 388 | 35.83 | 280 | 33.10 | 98 | 45.37 |
| **Other** | 375 | 34.63 | 329 | 38.89 | 46 | 21.30 |
| **TOTAL** | 1083 | 100.00 | 846 | 100.00 | 216 | 100.00 |

**Table S6: Crude and age-standardised rates of TBI-related mortality among residents in European countries by cause of injury, both sexes combined**

| **Country** | **Falls** | | | | **Traffic** | | | | **Suicide** | | | | **Violence** | | | | **Other** | | | |
| --- | --- | --- | --- | --- | --- | --- | --- | --- | --- | --- | --- | --- | --- | --- | --- | --- | --- | --- | --- | --- |
|  | **Crude Rate^1^** | **Standard. Rate^2^** | **LCI** | **UCI** | **Crude Rate^1^** | **Standard. Rate^2^** | **LCI** | **UCI** | **Crude Rate^1^** | **Standard. Rate^2^** | **LCI** | **UCI** | **Crude Rate^1^** | **Standard. Rate^2^** | **LCI** | **UCI** | **Crude Rate^1^** | **Standard. Rate^2^** | **LCI** | **UCI** |
|  |  |  |  |  |  |  |  |  |  |  |  |  |  |  |  |  |  |  |  |  |
| **AT** | 5.14 | 5.26 | 4.79 | 5.78 | 1.06 | 1.05 | 0.85 | 1.29 | 1.89 | 1.91 | 1.63 | 2.23 | 0.14 | 0.14 | 0.07 | 0.24 | 3.40 | 3.49 | 3.10 | 3.91 |
| **BE** | 6.04 | 6.20 | 5.74 | 6.69 | 2.08 | 2.10 | 1.84 | 2.39 | 1.16 | 1.19 | 0.99 | 1.41 | 0.24 | 0.24 | 0.16 | 0.35 | 1.78 | 1.83 | 1.59 | 2.11 |
| **BG** | 2.59 | 2.56 | 2.20 | 2.96 | 2.81 | 2.80 | 2.43 | 3.22 | 0.68 | 0.66 | 0.49 | 0.88 | 0.46 | 0.45 | 0.31 | 0.64 | 2.55 | 2.54 | 2.18 | 2.95 |
| **CY** | 1.42 | 2.03 | 1.03 | 3.65 | 2.60 | 2.81 | 1.73 | 4.42 | 1.06 | 0.99 | 0.45 | 2.09 | 0.35 | 0.42 | 0.09 | 1.41 | 0.83 | 1.12 | 0.43 | 2.45 |
| **CZ** | 3.50 | 3.74 | 3.37 | 4.15 | 3.08 | 3.09 | 2.76 | 3.45 | 1.90 | 1.91 | 1.65 | 2.20 | 0.32 | 0.32 | 0.22 | 0.45 | 5.22 | 5.63 | 5.16 | 6.13 |
| **DE** | 5.89 | 5.51 | 5.35 | 5.66 | 1.49 | 1.45 | 1.37 | 1.54 | 1.15 | 1.09 | 1.03 | 1.17 | 0.12 | 0.12 | 0.10 | 0.15 | 1.20 | 1.14 | 1.07 | 1.21 |
| **DK** | 2.64 | 2.87 | 2.42 | 3.37 | 1.90 | 1.92 | 1.58 | 2.33 | 1.06 | 1.07 | 0.82 | 1.38 | 0.23 | 0.23 | 0.12 | 0.40 | 0.49 | 0.52 | 0.35 | 0.76 |
| **EE** | 5.93 | 6.05 | 4.78 | 7.56 | 2.74 | 2.75 | 1.92 | 3.82 | 1.37 | 1.37 | 0.81 | 2.18 | 0.91 | 0.92 | 0.47 | 1.61 | 2.58 | 2.61 | 1.81 | 3.66 |
| **FI** | 11.57 | 11.45 | 10.57 | 12.37 | 2.19 | 2.19 | 1.81 | 2.61 | 2.66 | 2.68 | 2.27 | 3.16 | 0.42 | 0.43 | 0.27 | 0.65 | 1.77 | 1.77 | 1.43 | 2.16 |
| **GR** | 2.41 | 2.22 | 1.96 | 2.51 | 2.31 | 2.31 | 2.04 | 2.62 | 0.86 | 0.84 | 0.67 | 1.03 | 0.21 | 0.21 | 0.13 | 0.31 | 1.90 | 1.77 | 1.54 | 2.04 |
| **HR** | 6.42 | 6.90 | 6.10 | 7.80 | 2.35 | 2.32 | 1.89 | 2.84 | 2.09 | 2.08 | 1.67 | 2.58 | 0.33 | 0.33 | 0.18 | 0.56 | 0.97 | 1.01 | 0.73 | 1.39 |
| **HU** | 6.82 | 7.35 | 6.80 | 7.94 | 3.45 | 3.43 | 3.08 | 3.83 | 0.97 | 0.94 | 0.76 | 1.16 | 0.51 | 0.51 | 0.38 | 0.68 | 0.95 | 0.98 | 0.79 | 1.21 |
| **CH** | 7.03 | 7.36 | 6.77 | 7.98 | 1.11 | 1.13 | 0.91 | 1.38 | 0.68 | 0.69 | 0.52 | 0.89 | 0.07 | 0.08 | 0.03 | 0.17 | 1.10 | 1.13 | 0.91 | 1.40 |
| **IE** | 2.64 | 3.66 | 3.03 | 4.38 | 0.89 | 0.95 | 0.68 | 1.31 | 0.36 | 0.39 | 0.22 | 0.65 | 0.19 | 0.18 | 0.08 | 0.39 | 0.51 | 0.62 | 0.39 | 0.95 |
| **IS** | 2.42 | 3.14 | 1.34 | 6.29 | 0.91 | 0.73 | 0.15 | 2.62 | 0.60 | 0.51 | 0.06 | 2.32 | 0.00 | 0.00 | 0.00 | 0.00 | 0.91 | 1.29 | 0.27 | 3.78 |
| **IT** | 3.05 | 2.63 | 2.52 | 2.76 | 1.83 | 1.79 | 1.69 | 1.90 | 0.93 | 0.87 | 0.80 | 0.94 | 0.18 | 0.17 | 0.14 | 0.21 | 1.91 | 1.66 | 1.57 | 1.76 |
| **LT** | 7.50 | 7.64 | 6.66 | 8.73 | 2.48 | 2.43 | 1.90 | 3.07 | 0.62 | 0.61 | 0.36 | 0.97 | 1.48 | 1.46 | 1.06 | 1.97 | 6.02 | 6.03 | 5.17 | 7.00 |
| **LU** | 4.56 | 5.77 | 3.75 | 8.51 | 1.76 | 1.94 | 0.91 | 3.68 | 1.05 | 1.01 | 0.37 | 2.34 | 0.18 | 0.17 | 0.00 | 1.15 | 1.40 | 1.69 | 0.72 | 3.41 |
| **LV** | 5.82 | 5.83 | 4.81 | 7.01 | 2.43 | 2.41 | 1.78 | 3.21 | 0.96 | 0.95 | 0.57 | 1.49 | 1.47 | 1.47 | 0.98 | 2.12 | 5.56 | 5.53 | 4.55 | 6.68 |
| **MT** | 2.02 | 2.07 | 0.94 | 4.11 | 0.67 | 0.65 | 0.13 | 2.19 | 0.22 | 0.19 | 0.00 | 1.50 | 0.00 | 0.00 | 0.00 | 0.00 | 0.00 | 0.00 | 0.00 | 0.00 |
| **NL** | 5.15 | 5.75 | 5.37 | 6.14 | 1.88 | 1.94 | 1.73 | 2.17 | 0.85 | 0.85 | 0.72 | 1.01 | 0.21 | 0.21 | 0.15 | 0.30 | 1.16 | 1.27 | 1.09 | 1.46 |
| **NO** | 4.18 | 4.78 | 4.17 | 5.47 | 0.66 | 0.67 | 0.46 | 0.94 | 1.46 | 1.54 | 1.22 | 1.94 | 0.12 | 0.12 | 0.05 | 0.28 | 0.67 | 0.72 | 0.50 | 1.00 |
| **PT** | 4.30 | 4.11 | 3.74 | 4.51 | 2.37 | 2.33 | 2.05 | 2.64 | 1.58 | 1.52 | 1.30 | 1.78 | 0.32 | 0.31 | 0.21 | 0.44 | 2.97 | 2.86 | 2.55 | 3.20 |
| **RO** | 3.78 | 3.96 | 3.68 | 4.26 | 3.81 | 3.83 | 3.56 | 4.12 | 0.12 | 0.11 | 0.07 | 0.17 | 0.49 | 0.50 | 0.40 | 0.61 | 2.33 | 2.38 | 2.16 | 2.61 |
| **RS** | 3.13 | 3.38 | 2.94 | 3.88 | 2.89 | 2.85 | 2.48 | 3.29 | 1.69 | 1.66 | 1.38 | 2.00 | 0.48 | 0.47 | 0.33 | 0.68 | 2.88 | 2.99 | 2.59 | 3.45 |
| **SE** | 5.01 | 4.93 | 4.50 | 5.39 | 1.08 | 1.08 | 0.88 | 1.31 | 1.35 | 1.37 | 1.14 | 1.62 | 0.23 | 0.23 | 0.15 | 0.35 | 2.37 | 2.33 | 2.04 | 2.65 |
| **SI** | 5.23 | 5.62 | 4.61 | 6.79 | 1.07 | 1.06 | 0.66 | 1.62 | 1.36 | 1.38 | 0.91 | 2.00 | 0.05 | 0.05 | 0.00 | 0.30 | 1.07 | 1.07 | 0.67 | 1.63 |
| **SK** | 5.60 | 6.67 | 5.92 | 7.50 | 2.45 | 2.46 | 2.05 | 2.94 | 1.07 | 1.13 | 0.85 | 1.48 | 0.18 | 0.20 | 0.09 | 0.40 | 5.13 | 5.90 | 5.20 | 6.67 |
| **TR** | 0.83 | 1.80 | 1.65 | 1.96 | 2.22 | 2.68 | 2.54 | 2.83 | 0.27 | 0.28 | 0.24 | 0.33 | 0.17 | 0.16 | 0.13 | 0.20 | 0.71 | 0.75 | 0.69 | 0.83 |
| **UK** | 4.49 | 4.83 | 4.65 | 5.00 | 0.72 | 0.72 | 0.65 | 0.79 | 0.24 | 0.25 | 0.21 | 0.29 | 0.03 | 0.04 | 0.02 | 0.05 | 1.19 | 1.26 | 1.17 | 1.35 |

*^1^per 100,000 population; ^2^per 100,000 population. age-standardised rate using the European standard population 2013*

*AT. Austria; BE. Belgium; BG. Bulgaria; CY. Cyprus; CZ. Czechia; DE. Germany; DK. Denmark; EE. Estonia; FI. Finland; GR. Greece; HR. Croatia; HU. Hungary; CH. Switzerland; IE. Ireland; IS. Island; IT. Italy; LT. Lithuania; LU. Luxemburg; LV. Latvia; MT. Malta; NL. Netherlands; NO. Norway; PT. Portugal; RO. Romania; RS. Serbia; SE. Sweden; SI. Slovenia; SK. Slovakia; TR. Turkey; UK. United Kingdom*

**Table S7: Crude and age-standardised rates of TBI-related mortality among non-residents in European countries by cause of injury, both sexes combined**

| **Country** | **Falls** | | | | **Traffic** | | | | **Suicide** | | | | **Violence** | | | | **Other** | | | |
| --- | --- | --- | --- | --- | --- | --- | --- | --- | --- | --- | --- | --- | --- | --- | --- | --- | --- | --- | --- | --- |
|  | **Crude Rate^1^** | **Adjusted Rate^2^** | **LCI** | **UCI** | **Crude Rate^1^** | **Adjusted Rate^2^** | **LCI** | **UCI** | **Crude Rate^1^** | **Adjusted Rate^2^** | **LCI** | **UCI** | **Crude Rate^1^** | **Adjusted Rate^2^** | **LCI** | **UCI** | **Crude Rate^1^** | **Adjusted Rate^2^** | **LCI** | **UCI** |
|  |  |  |  |  |  |  |  |  |  |  |  |  |  |  |  |  |  |  |  |  |
| **AT** | 0.34 | 0.34 | 0.23 | 0.49 | 0.07 | 0.07 | 0.03 | 0.16 | 0.02 | 0.02 | 0.00 | 0.09 | 0.02 | 0.02 | 0.00 | 0.09 | 0.16 | 0.16 | 0.09 | 0.27 |
| **BE** | 0.08 | 0.08 | 0.04 | 0.16 | 0.15 | 0.15 | 0.09 | 0.24 | 0.04 | 0.04 | 0.01 | 0.09 | 0.04 | 0.04 | 0.01 | 0.09 | 0.01 | 0.01 | 0.00 | 0.05 |
| **BG** | 0.01 | 0.01 | 0.00 | 0.09 | 0.03 | 0.03 | 0.00 | 0.11 | 0.03 | 0.03 | 0.00 | 0.12 | 0.00 | 0.00 | 0.00 | 0.00 | 0.04 | 0.04 | 0.01 | 0.13 |
| **CY** | 0.00 | 0.00 | 0.00 | 0.00 | 0.47 | 0.44 | 0.12 | 1.37 | 0.00 | 0.00 | 0.00 | 0.00 | 0.00 | 0.00 | 0.00 | 0.00 | 0.00 | 0.00 | 0.00 | 0.00 |
| **CZ** | 0.10 | 0.11 | 0.05 | 0.20 | 0.16 | 0.16 | 0.09 | 0.26 | 0.05 | 0.05 | 0.02 | 0.12 | 0.01 | 0.01 | 0.00 | 0.06 | 0.08 | 0.07 | 0.03 | 0.15 |
| **DE** | 0.02 | 0.02 | 0.01 | 0.04 | 0.07 | 0.07 | 0.05 | 0.09 | 0.01 | 0.01 | 0.00 | 0.02 | 0.00 | 0.00 | 0.00 | 0.01 | 0.02 | 0.02 | 0.01 | 0.03 |
| **DK** | 0.04 | 0.04 | 0.00 | 0.14 | 0.04 | 0.04 | 0.00 | 0.14 | 0.00 | 0.00 | 0.00 | 0.00 | 0.00 | 0.00 | 0.00 | 0.00 | 0.02 | 0.02 | 0.00 | 0.11 |
| **EE** | 0.00 | 0.00 | 0.00 | 0.00 | 0.08 | 0.08 | 0.00 | 0.45 | 0.00 | 0.00 | 0.00 | 0.00 | 0.00 | 0.00 | 0.00 | 0.00 | 0.08 | 0.08 | 0.00 | 0.45 |
| **FI** | 0.02 | 0.02 | 0.00 | 0.11 | 0.00 | 0.00 | 0.00 | 0.00 | 0.00 | 0.00 | 0.00 | 0.00 | 0.04 | 0.04 | 0.00 | 0.13 | 0.04 | 0.04 | 0.00 | 0.13 |
| **GR** | 0.05 | 0.04 | 0.01 | 0.11 | 0.11 | 0.11 | 0.06 | 0.20 | 0.00 | 0.00 | 0.00 | 0.00 | 0.04 | 0.04 | 0.01 | 0.10 | 0.07 | 0.07 | 0.03 | 0.14 |
| **HR** | 0.17 | 0.17 | 0.07 | 0.37 | 0.17 | 0.16 | 0.07 | 0.35 | 0.05 | 0.05 | 0.01 | 0.19 | 0.00 | 0.00 | 0.00 | 0.00 | 0.00 | 0.00 | 0.00 | 0.00 |
| **HU** | 0.03 | 0.03 | 0.01 | 0.10 | 0.23 | 0.23 | 0.14 | 0.35 | 0.01 | 0.01 | 0.00 | 0.07 | 0.00 | 0.00 | 0.00 | 0.00 | 0.01 | 0.01 | 0.00 | 0.07 |
| **CH** | 0.25 | 0.26 | 0.16 | 0.39 | 0.16 | 0.15 | 0.08 | 0.26 | 0.01 | 0.01 | 0.00 | 0.07 | 0.01 | 0.01 | 0.00 | 0.07 | 0.04 | 0.04 | 0.01 | 0.11 |
| **IE** | 0.06 | 0.09 | 0.02 | 0.29 | 0.06 | 0.06 | 0.01 | 0.23 | 0.00 | 0.00 | 0.00 | 0.00 | 0.02 | 0.03 | 0.00 | 0.19 | 0.00 | 0.00 | 0.00 | 0.00 |
| **IS** | 0.00 | 0.00 | 0.00 | 0.00 | 0.00 | 0.00 | 0.00 | 0.00 | 0.00 | 0.00 | 0.00 | 0.00 | 0.00 | 0.00 | 0.00 | 0.00 | 0.00 | 0.00 | 0.00 | 0.00 |
| **IT** | 0.06 | 0.06 | 0.04 | 0.08 | 0.10 | 0.10 | 0.08 | 0.13 | 0.01 | 0.01 | 0.00 | 0.02 | 0.02 | 0.03 | 0.01 | 0.04 | 0.02 | 0.02 | 0.01 | 0.03 |
| **LT** | 0.00 | 0.00 | 0.00 | 0.00 | 0.03 | 0.03 | 0.00 | 0.21 | 0.00 | 0.00 | 0.00 | 0.00 | 0.00 | 0.00 | 0.00 | 0.00 | 0.03 | 0.03 | 0.00 | 0.21 |
| **LU** | 0.35 | 0.32 | 0.04 | 1.38 | 0.53 | 0.49 | 0.10 | 1.63 | 0.00 | 0.00 | 0.00 | 0.00 | 0.00 | 0.00 | 0.00 | 0.00 | 0.00 | 0.00 | 0.00 | 0.00 |
| **LV** | 0.05 | 0.05 | 0.00 | 0.30 | 0.00 | 0.00 | 0.00 | 0.00 | 0.00 | 0.00 | 0.00 | 0.00 | 0.00 | 0.00 | 0.00 | 0.00 | 0.00 | 0.00 | 0.00 | 0.00 |
| **MT** | 0.00 | 0.00 | 0.00 | 0.00 | 0.00 | 0.00 | 0.00 | 0.00 | 0.00 | 0.00 | 0.00 | 0.00 | 0.00 | 0.00 | 0.00 | 0.00 | 0.00 | 0.00 | 0.00 | 0.00 |
| **NL** | 0.08 | 0.08 | 0.05 | 0.14 | 0.14 | 0.14 | 0.09 | 0.21 | 0.05 | 0.05 | 0.02 | 0.10 | 0.02 | 0.02 | 0.00 | 0.05 | 0.02 | 0.02 | 0.00 | 0.06 |
| **NO** | 0.12 | 0.12 | 0.04 | 0.26 | 0.13 | 0.13 | 0.05 | 0.28 | 0.02 | 0.02 | 0.00 | 0.11 | 0.04 | 0.04 | 0.00 | 0.15 | 0.13 | 0.13 | 0.05 | 0.28 |
| **PT** | 0.09 | 0.08 | 0.04 | 0.16 | 0.09 | 0.09 | 0.04 | 0.17 | 0.00 | 0.00 | 0.00 | 0.00 | 0.00 | 0.00 | 0.00 | 0.00 | 0.04 | 0.04 | 0.01 | 0.10 |
| **RO** | 0.02 | 0.02 | 0.00 | 0.05 | 0.02 | 0.02 | 0.01 | 0.06 | 0.00 | 0.00 | 0.00 | 0.00 | 0.00 | 0.00 | 0.00 | 0.00 | 0.01 | 0.01 | 0.00 | 0.04 |
| **RS** | 0.00 | 0.00 | 0.00 | 0.00 | 0.00 | 0.00 | 0.00 | 0.00 | 0.00 | 0.00 | 0.00 | 0.00 | 0.00 | 0.00 | 0.00 | 0.00 | 0.00 | 0.00 | 0.00 | 0.00 |
| **SE** | 0.09 | 0.09 | 0.04 | 0.18 | 0.11 | 0.11 | 0.06 | 0.20 | 0.01 | 0.01 | 0.00 | 0.06 | 0.04 | 0.04 | 0.01 | 0.11 | 0.00 | 0.00 | 0.00 | 0.00 |
| **SI** | 0.00 | 0.00 | 0.00 | 0.00 | 0.00 | 0.00 | 0.00 | 0.00 | 0.00 | 0.00 | 0.00 | 0.00 | 0.00 | 0.00 | 0.00 | 0.00 | 0.00 | 0.00 | 0.00 | 0.00 |
| **SK** | 0.07 | 0.08 | 0.02 | 0.25 | 0.07 | 0.07 | 0.02 | 0.22 | 0.00 | 0.00 | 0.00 | 0.00 | 0.00 | 0.00 | 0.00 | 0.00 | 0.02 | 0.02 | 0.00 | 0.17 |
| **TR** | 0.02 | 0.02 | 0.01 | 0.04 | 0.12 | 0.11 | 0.09 | 0.15 | 0.00 | 0.00 | 0.00 | 0.02 | 0.01 | 0.01 | 0.00 | 0.02 | 0.36 | 0.31 | 0.27 | 0.35 |
| **UK** | 0.02 | 0.02 | 0.01 | 0.03 | 0.01 | 0.01 | 0.01 | 0.03 | 0.00 | 0.00 | 0.00 | 0.01 | 0.00 | 0.00 | 0.00 | 0.00 | 0.01 | 0.01 | 0.00 | 0.02 |

*^1^per 100,000 population; ^2^per 100,000 population. age-standardised rate using the European standard population 2013*

*AT. Austria; BE. Belgium; BG. Bulgaria; CY. Cyprus; CZ. Czechia; DE. Germany; DK. Denmark; EE. Estonia; FI. Finland; GR. Greece; HR. Croatia; HU. Hungary; CH. Switzerland; IE. Ireland; IS. Island; IT. Italy; LT. Lithuania; LU. Luxemburg; LV. Latvia; MT. Malta; NL. Netherlands; NO. Norway; PT. Portugal; RO. Romania; RS. Serbia; SE. Sweden; SI. Slovenia; SK. Slovakia; TR. Turkey; UK. United Kingdom*

**Table S8: Extrapolation of the observed age-standardised TBI-related mortality rates among non-residents to the populations of the EU and Europe as a continent**

| **Observed**  **Age-standardised Rate** | **95% Confidence Interval** | **Extrapolated Estimates** | | | | | |
| --- | --- | --- | --- | --- | --- | --- | --- |
|  |  | **EU-27** | | **EU-28** | | **Europe** | |
|  |  | **Estimate** | **95% Confidence Interval** | **Estimate** | **95% Confidence Interval** | **Estimate** | **95% Confidence Interval** |
| 0.2 | 0.1–0.36 | 891 | 445–1603 | 1022 | 511–1840 | 1488 | 744–2768 |

*For the estimations the average population counts for 2015-2019 were used: 445,331,815 for the EU-27, 511,131,268 for the EU-28, and 743,779,091 for Europe as a continent*

*Sources of data:*

*Eurostat:* [*https://ec.europa.eu/eurostat/databrowser/view/demo_pjan/default/table?lang=en*](https://ec.europa.eu/eurostat/databrowser/view/demo_pjan/default/table?lang=en)

*UN Department of Economic and Social Affairs Population Division:* [*https://population.un.org/wpp/Download/Standard/MostUsed/*](https://population.un.org/wpp/Download/Standard/MostUsed/)

**Table S9: Distribution of causes of death among the range of ICD-10 codes used for case definition, by country.**

| **Country** | **S00**  **Superficial injury of head** | | **S01**  **Open wound of head** | | **S02**  **Fracture of skull and facial bones** | | **S03**  **Dislocation and sprain of joints and ligaments of head** | | **S04**  **Injury of cranial nerve** | | **S05**  **Injury of eye and orbit** | | **S06**  **Intracranial injury** | | **S07**  **Crushing injury of head** | | **S08**  **Avulsion and traumatic amputation of part of head** | | **S09**  **Other and unspecified injuries of head** | | **T90**  **Sequelae of injuries of head** | | **Total** | |
| --- | --- | --- | --- | --- | --- | --- | --- | --- | --- | --- | --- | --- | --- | --- | --- | --- | --- | --- | --- | --- | --- | --- | --- | --- |
|  | **N** | **%** | **N** | **%** | **N** | **%** | **N** | **%** | **N** | **%** | **N** | **%** | **N** | **%** | **N** | **%** | **N** | **%** | **N** | **%** | **N** | **%** | **N** | **%** |
| **AT** | 10 | 0,9 | 145 | 13,7 | 89 | 8,4 | 0 | 0,0 | 0 | 0,0 | 0 | 0,0 | 757 | 71,6 | 0 | 0,0 | 0 | 0,0 | 9 | 0,9 | 48 | 4,5 | 1058 | 100,0 |
| **BE** | 3 | 0,2 | 21 | 1,6 | 47 | 3,6 | 0 | 0,0 | 1 | 0,1 | 0 | 0,0 | 1005 | 76,7 | 2 | 0,2 | 0 | 0,0 | 220 | 16,8 | 11 | 0,8 | 1310 | 100,0 |
| **BG** | 1 | 0,2 | 28 | 4,2 | 68 | 10,3 | 0 | 0,0 | 14 | 2,1 | 0 | 0,0 | 436 | 66,0 | 14 | 2,1 | 1 | 0,2 | 71 | 10,7 | 28 | 4,2 | 661 | 100,0 |
| **CY** | 0 | 0,0 | 0 | 0,0 | 0 | 0,0 | 0 | 0,0 | 0 | 0,0 | 0 | 0,0 | 55 | 96,5 | 0 | 0,0 | 0 | 0,0 | 2 | 3,5 | 0 | 0,0 | 57 | 100,0 |
| **CZ** | 0 | 0,0 | 15 | 1,0 | 35 | 2,3 | 0 | 0,0 | 0 | 0,0 | 1 | 0,1 | 1341 | 88,3 | 54 | 3,6 | 2 | 0,1 | 41 | 2,7 | 31 | 2,0 | 1519 | 100,0 |
| **DE** | 46 | 0,6 | 501 | 6,2 | 563 | 6,9 | 28 | 0,3 | 12 | 0,1 | 27 | 0,3 | 6309 | 77,6 | 39 | 0,5 | 35 | 0,4 | 467 | 5,7 | 125 | 1,5 | 8125 | 100,0 |
| **DK** | 0 | 0,0 | 4 | 1,1 | 34 | 9,3 | 0 | 0,0 | 0 | 0,0 | 0 | 0,0 | 230 | 63,2 | 69 | 19,0 | 0 | 0,0 | 24 | 6,6 | 3 | 0,8 | 364 | 100,0 |
| **EE** | 0 | 0,0 | 0 | 0,0 | 7 | 3,9 | 0 | 0,0 | 0 | 0,0 | 0 | 0,0 | 152 | 84,4 | 19 | 10,6 | 0 | 0,0 | 2 | 1,1 | 0 | 0,0 | 180 | 100,0 |
| **FI** | 0 | 0,0 | 5 | 0,5 | 33 | 3,2 | 0 | 0,0 | 0 | 0,0 | 0 | 0,0 | 766 | 74,7 | 75 | 7,3 | 3 | 0,3 | 100 | 9,8 | 43 | 4,2 | 1025 | 100,0 |
| **GR** | 0 | 0,0 | 1 | 0,1 | 79 | 9,2 | 0 | 0,0 | 0 | 0,0 | 0 | 0,0 | 742 | 86,1 | 1 | 0,1 | 0 | 0,0 | 39 | 4,5 | 0 | 0,0 | 862 | 100,0 |
| **HR** | 0 | 0,0 | 89 | 16,9 | 77 | 14,6 | 0 | 0,0 | 0 | 0,0 | 0 | 0,0 | 315 | 59,7 | 12 | 2,3 | 1 | 0,2 | 30 | 5,7 | 4 | 0,8 | 528 | 100,0 |
| **HU** | 1 | 0,1 | 1 | 0,1 | 50 | 3,9 | 0 | 0,0 | 0 | 0,0 | 0 | 0,0 | 1178 | 92,2 | 1 | 0,1 | 0 | 0,0 | 40 | 3,1 | 7 | 0,5 | 1278 | 100,0 |
| **CH** | 6 | 0,7 | 3 | 0,3 | 53 | 6,1 | 0 | 0,0 | 1 | 0,1 | 0 | 0,0 | 779 | 90,0 | 1 | 0,1 | 0 | 0,0 | 23 | 2,7 | 0 | 0,0 | 866 | 100,0 |
| **IE** | 0 | 0,0 | 18 | 8,1 | 12 | 5,4 | 0 | 0,0 | 0 | 0,0 | 0 | 0,0 | 147 | 65,9 | 1 | 0,4 | 0 | 0,0 | 42 | 18,8 | 3 | 1,3 | 223 | 100,0 |
| **IS** | 0 | 0,0 | 0 | 0,0 | 0 | 0,0 | 0 | 0,0 | 0 | 0,0 | 0 | 0,0 | 14 | 87,5 | 1 | 6,3 | 0 | 0,0 | 1 | 6,3 | 0 | 0,0 | 16 | 100,0 |
| **IT** | 6 | 0,1 | 185 | 3,8 | 910 | 18,5 | 0 | 0,0 | 0 | 0,0 | 5 | 0,1 | 3538 | 71,9 | 9 | 0,2 | 0 | 0,0 | 141 | 2,9 | 129 | 2,6 | 4918 | 100,0 |
| **LT** | 0 | 0,0 | 5 | 0,9 | 54 | 10,2 | 0 | 0,0 | 0 | 0,0 | 1 | 0,2 | 274 | 52,0 | 2 | 0,4 | 0 | 0,0 | 170 | 32,3 | 22 | 4,2 | 527 | 100,0 |
| **LU** | 0 | 0,0 | 6 | 10,7 | 2 | 3,6 | 0 | 0,0 | 0 | 0,0 | 0 | 0,0 | 45 | 80,4 | 0 | 0,0 | 1 | 1,8 | 0 | 0,0 | 2 | 3,6 | 56 | 100,0 |
| **LV** | 0 | 0,0 | 7 | 2,2 | 100 | 31,1 | 0 | 0,0 | 0 | 0,0 | 0 | 0,0 | 104 | 32,3 | 2 | 0,6 | 0 | 0,0 | 91 | 28,3 | 18 | 5,6 | 322 | 100,0 |
| **MT** | 0 | 0,0 | 0 | 0,0 | 4 | 30,8 | 0 | 0,0 | 0 | 0,0 | 0 | 0,0 | 6 | 46,2 | 0 | 0,0 | 0 | 0,0 | 3 | 23,1 | 0 | 0,0 | 13 | 100,0 |
| **NL** | 6 | 0,4 | 50 | 3,1 | 159 | 9,8 | 0 | 0,0 | 0 | 0,0 | 2 | 0,1 | 1127 | 69,7 | 17 | 1,1 | 4 | 0,2 | 149 | 9,2 | 105 | 6,5 | 1617 | 100,0 |
| **NO** | 0 | 0,0 | 0 | 0,0 | 21 | 5,4 | 0 | 0,0 | 0 | 0,0 | 0 | 0,0 | 251 | 64,2 | 8 | 2,0 | 0 | 0,0 | 100 | 25,6 | 11 | 2,8 | 391 | 100,0 |
| **PT** | 0 | 0,0 | 3 | 0,2 | 147 | 12,1 | 0 | 0,0 | 0 | 0,0 | 1 | 0,1 | 898 | 73,8 | 0 | 0,0 | 0 | 0,0 | 136 | 11,2 | 33 | 2,7 | 1217 | 100,0 |
| **RO** | 0 | 0,0 | 37 | 1,8 | 348 | 16,6 | 0 | 0,0 | 0 | 0,0 | 3 | 0,1 | 1440 | 68,9 | 107 | 5,1 | 1 | 0,0 | 158 | 7,6 | 0 | 0,0 | 2091 | 100,0 |
| **RS** | 0 | 0,0 | 74 | 9,4 | 148 | 18,9 | 4 | 0,5 | 0 | 0,0 | 0 | 0,0 | 499 | 63,6 | 15 | 1,9 | 1 | 0,1 | 42 | 5,4 | 2 | 0,3 | 785 | 100,0 |
| **SE** | 0 | 0,0 | 10 | 1,0 | 12 | 1,2 | 0 | 0,0 | 0 | 0,0 | 0 | 0,0 | 887 | 87,9 | 41 | 4,1 | 0 | 0,0 | 26 | 2,6 | 33 | 3,3 | 1009 | 100,0 |
| **SI** | 0 | 0,0 | 0 | 0,0 | 10 | 5,5 | 0 | 0,0 | 0 | 0,0 | 0 | 0,0 | 80 | 44,2 | 12 | 6,6 | 0 | 0,0 | 69 | 38,1 | 10 | 5,5 | 181 | 100,0 |
| **SK** | 2 | 0,3 | 14 | 1,8 | 147 | 18,6 | 1 | 0,1 | 0 | 0,0 | 0 | 0,0 | 535 | 67,6 | 50 | 6,3 | 4 | 0,5 | 31 | 3,9 | 8 | 1,0 | 792 | 100,0 |
| **TR** | 2 | 0,1 | 9 | 0,2 | 543 | 14,7 | 0 | 0,0 | 0 | 0,0 | 0 | 0,0 | 1813 | 49,2 | 45 | 1,2 | 2 | 0,1 | 1255 | 34,1 | 14 | 0,4 | 3683 | 100,0 |
| **UK** | 1 | 0,0 | 106 | 2,4 | 71 | 1,6 | 0 | 0,0 | 1 | 0,0 | 0 | 0,0 | 3084 | 70,5 | 5 | 0,1 | 1 | 0,0 | 1028 | 23,5 | 76 | 1,7 | 4373 | 100,0 |
| **Total** | **84** | **0,2** | **1337** | **3,3** | **3823** | **9,5** | **33** | **0,1** | **29** | **0,1** | **40** | **0,1** | **28807** | **71,9** | **602** | **1,5** | **56** | **0,1** | **4510** | **11,3** | **766** | **1,9** | **40047** | **100,0** |
